# EXPANDS: expanding ploidy and allele frequency on nested subpopulations

**DOI:** 10.1093/bioinformatics/btt622

**Published:** 2013-10-30

**Authors:** Noemi Andor, Julie V. Harness, Sabine Müller, Hans W. Mewes, Claudia Petritsch

**Affiliations:** ^1^Department of Neurological Surgery, University of California San Francisco, San Francisco, CA 94143, USA, ^2^Institute of Bioinformatics and Systems Biology, Helmholtz Zentrum München, German Research Center for Environmental Health, 85764 Neuherberg, Germany, ^3^Brain Tumor Research Center, University of California San Francisco, San Francisco, CA 94158, USA, ^4^Department of Neurology, University of California San Francisco, San Francisco, CA 94143, USA, ^5^Department of Pediatrics, University of California San Francisco, San Francisco, CA 94143, USA, ^6^Chair of Genome Oriented Bioinformatics, Center of Life and Food Science, Freising-Weihenstephan, Technische Universität München, 80333, Munich, Germany, ^7^Helen Diller Family Comprehensive Cancer Center, University of California San Francisco, San Francisco, CA 94158 and ^8^Eli and Edythe Broad Center of Regeneration Medicine and Stem Cell Research, University of California San Francisco, San Francisco, CA 94143, USA

## Abstract

**Motivation:** Several cancer types consist of multiple genetically and phenotypically distinct subpopulations. The underlying mechanism for this intra-tumoral heterogeneity can be explained by the clonal evolution model, whereby growth advantageous mutations cause the expansion of cancer cell subclones. The recurrent phenotype of many cancers may be a consequence of these coexisting subpopulations responding unequally to therapies. Methods to computationally infer tumor evolution and subpopulation diversity are emerging and they hold the promise to improve the understanding of genetic and molecular determinants of recurrence.

**Results:** To address cellular subpopulation dynamics within human tumors, we developed a bioinformatic method, EXPANDS. It estimates the proportion of cells harboring specific mutations in a tumor. By modeling cellular frequencies as probability distributions, EXPANDS predicts mutations that accumulate in a cell before its clonal expansion. We assessed the performance of EXPANDS on one whole genome sequenced breast cancer and performed SP analyses on 118 glioblastoma multiforme samples obtained from TCGA. Our results inform about the extent of subclonal diversity in primary glioblastoma, subpopulation dynamics during recurrence and provide a set of candidate genes mutated in the most well-adapted subpopulations. In summary, EXPANDS predicts tumor purity and subclonal composition from sequencing data.

**Availability and implementation:** EXPANDS is available for download at http://code.google.com/p/expands (matlab version - used in this manuscript) and http://cran.r-project.org/web/packages/expands (R version).

**Contact**: claudia.petritsch@ucsf.edu

**Supplementary information:** Supplementary data are available at *Bioinformatics* online.

## 1 INTRODUCTION

The clonal evolution model initially proposed by Peter C. Nowell posits that a single cell of origin or ‘clone’ that underwent transformation from a normal to a cancerous state undergoes clonal expansion and through successive acquisition of mutations generates genetically diverse subclones (Nowell, 1976). Subsequent pressure from the cancer microenvironment selects the fittest subclone(s), driving their expansion into subpopulations (SPs). As a consequence, the resulting neoplasm is composed of multiple genetically diverse SPs that are best adapted to their microenvironment.

Cancer therapies are hypothesized to eliminate some but not all SPs within a tumor, resulting in altered SP composition of tumors that recur post-treatment (Aktipis *et al.*, 2011; Merlo and Maley, 2010). A better understanding of the SP composition and dynamics of individual tumors is expected to have significant clinical implications especially for highly recurrent and therapy resistant tumors. Glioblastoma (GBM), a grade IV astrocytoma, is the most common CNS tumor in adults. Despite aggressive standard therapy, consisting of surgery, radiation and adjuvant chemotherapy with the DNA alkylating drug temozolomide (Stupp *et al.*, 2009), GBM invariably recur within months following initial diagnosis (Konishi *et al.*, 2012) and typically become resistant to the first line therapy they have been exposed (Fan *et al.*, 2010; Lomonaco *et al.*, 2009). An SP with stem-like properties, the cancer stem cells, is one potential culprit for recurrence and therapy resistance in GBM (Chen *et al.*, 2012; Schonberg *et al.*, 2013). GBM exhibit substantial subclonal diversity as evidenced by previous comparative genomic hybridization (CGH) analyses (Harada *et al.*, 1998), fluorescence *in situ* hybridization studies (Little *et al.*, 2012; Snuderl *et al.*, 2011; Szerlip *et al.*, 2012) and areal sampling of surgical tissue followed by molecular analyses (Sottoriva *et al.*, 2013). Moreover, heterogeneity within GBM is maintained by cross-talk between genetically distinct tumor cell SPs (Inda *et al.*, 2010). However, genome-wide analyses of intra-tumoral heterogeneity and the clonal cellular SP dynamics within GBM have yet to be performed.

Clonal architecture analyses as previously performed for acute lymphoblastic leukemia single cells (Anderson *et al.*, 2011; Navin *et al.*, 2011) are difficult to perform for solid tumors that are a tight mix of distinct SPs and various fractions of non-neoplastic cells. Knowing the purity of a tumor sample is important to determine sample quality and to adjust parameters during copy-number estimation and detection of somatic mutations. The advances in next-generation sequencing (NGS) technologies provide emerging tools for computational methods to overcome those hurdles. ABSOLUTE for example is an analytic method that predicts tumor purity and the distribution of mutations within the tumor from exome sequencing data (Carter *et al.*, 2012). The inferences made by ABSOLUTE do not include the number of tumor SPs and the size of each SP in the tumor bulk. These are, however, desirable parameters when quantifying genetic heterogeneity, an emerging biomarker for clinical outcome (Merlo *et al.*, 2010, Mroz *et al.*, 2013).

Shah *et al.* have developed another bioinformatic approach to predict the number and distribution of subclones from deep-sequenced selected mutated genes and to infer the clonal evolution of triple negative breast cancer (Shah *et al.*, 2012). By this method, two SPs with the same mutation in the region that was deep sequenced would be indistinguishable even if greatly divergent in other areas of the genome. This approach further relies on prior knowledge or hypotheses regarding genes of interest and does not allow for unbiased exploration of the cancer genome.

Nik-Zainal *et al.* modeled the observed patterns of clonal and subclonal mutations with a hierarchical Bayesian Dirichlet process (Nik-Zainal *et al.*, 2012). This approach was designed specifically for the mutations detected in the whole genome of a hypermutated breast cancer, sequenced at high coverage (188-fold). The authors inferred a plethora of information about the evolution and subclonal structure of this cancer genome by using various ways to look at the data as described in Nik-Zainal *et al.* (2012), yet their combined approaches are not available as one automated method.

The resolution of the aforementioned approaches increases with depth of coverage. We propose that the accuracy in the identification of SPs is dependent not only on depth of coverage but also on breadth of coverage, i.e. the fraction of nucleotides in the genome sampled by the assay. Here we intend to complement existing methods by pairing an unbiased genome-wide sequencing approach with a robust analytic algorithm.

We present Expanding Ploidy and Allele-frequency on Nested Subpopulations (EXPANDS), a method that characterizes coexisting SPs in a tumor using copy number and allele frequencies derived from exome- or whole genome sequencing input data. The model amplifies the statistical power to detect coexisting genotypes, by fully exploiting run-specific tradeoffs between depth of coverage and breadth of coverage. Our results indicate that EXPANDS is superior to ABSOLUTE in predicting tumor purity of highly heterogeneous tumor samples. In addition to tumor purity, EXPANDS predicts the number of clonal expansions, the size of the resulting SPs in the tumor bulk and the mutations specific to each SP. This information can be useful to identify candidate gene regulators of tumor growth and recurrence.

The article outline is as follows: in Section 2.1–2.2, we formulate the problem and describe the EXPANDS model. Section 2.3 describes the performance of EXPANDS on a simulated dataset, data obtained from one previously published whole genome sequenced breast cancer case and 118 TCGA GBM exome-sequenced samples. Section 3 shows how our approach results inform about SP composition in primary GBM and SP changes on recurrence and what genes are mutated in the most well-adapted SPs. Finally, in Section 4, we discuss limitations to our method and propose future directions for the approach of tumor mixture separation.

## 2 SYSTEM AND METHODS

### 2.1 Problem formulation

Given a set of somatic point mutations, 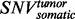, detected in a tumor sample and the copy number of the genomic segments in which the mutations are located, we aim to identify the number *N* of clonal expansions within the tumor, the relative size *f_i_* of the resulting SPs in the tumor bulk and the mutations habitant in each *SP_i_, i = {1…N}*. We assume that both, technical artifacts (sequencing errors, mapping errors) and germline polymorphisms, have been adequately filtered. First, we identify the number and size of SPs. Finally, we assign each 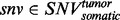 to an SP, *SP_i_*. The set of somatic mutations can be extended to contain loss of heterozygosity (LOH) sites for which the non-reference allele is overrepresented in the cancer cell relative to a normal cell. For tumors with a low number of somatic point mutations, the inclusion of LOH sites can provide a sufficient number of somatic events for the subsequent procedure.

### 2.2 EXPANDS model

**Fig. 1.**
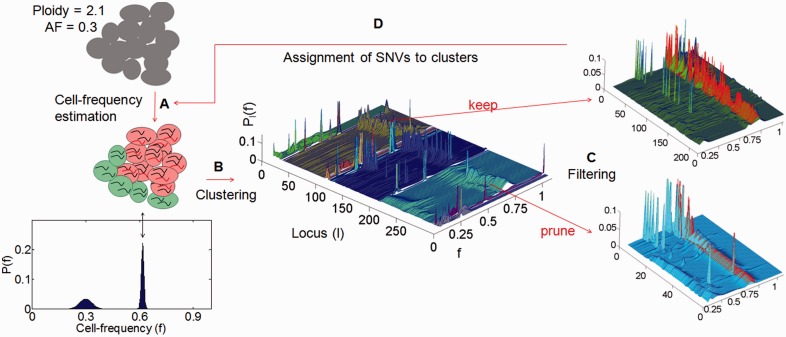
Graphical summary of the four major steps (**A–D**) of EXPANDS. Given a set of SNVs, EXPANDS predicts the number of clonal expansions in a tumor, the size of the resulting SPs in the tumor bulk and which SNVs accumulate in a cell before its clonal expansion. The copy number and allele frequency assigned to a SNV are measures of aggregate signals from many cells. (A) Cell frequency estimation. EXPANDS combines these two measurements to estimate what fraction of cells harbor the SNV. In this example, the observed AF (0.3) and copy number (2.1) can be explained either by a homozygous mutation, present in 30% of the cells or a heterozygous mutation, present in 60% of the cells. The cell-frequency probability P(f) is computed for each mutated locus separately. (B) Clustering. All SNVs are clustered based on their cell-frequency probability distributions. Each cluster is extended by members with similar distributions in an interval around the cluster-maxima. (C) Filtering. Clusters are pruned based on statistics within and outside the core region (interval around the cluster-maxima: highlighted in red). The blue cluster is pruned as peaks within the core region are low and do not significantly exceed peaks observed outside the core region. In contrast, the green cluster is kept as it has high and abundant peaks within and only a few peaks outside the core region. The number of remaining clusters denotes the number of predicted clonal expansions. Cell frequencies at cluster-maxima denote the predicted size of an SP in the tumor bulk. (D) Assignment of SNVs to clusters. Each SNV is assigned to one of the predicted clonal expansions, based on the cell frequency estimation computed in (A)

Tumor cells acquire novel mutations that distinguish them from other cells within the same tumor. With respect to the whole genome, there is no limit to how diverse a tumor cell population can become. However, with respect to a specific locus *l*, only a limited number of possible states exist, each characterized by 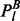
—the ploidy of the non-reference allele (B allele) and 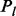
—the total ploidy of locus *l*. Given the stochastic nature of somatic events and the size of the human genome, it is unlikely that two independent driver-events of the same type will target the same genomic position in two different cells. Therefore, we assume that no more than two distinct cell types exist with respect to a specific locus *l*: cells in which the locus is in its mutated state and cells in which the locus is in its normal state, further denoted mutated cells and normal cells, respectively. Furthermore, we assume that multiple passenger mutations accumulate in a cell before a driver mutation causes a clonal expansion and thus, that each clonal expansion is marked by multiple mutations. These two assumptions are translated into the EXPANDS model in four main steps: cell frequency estimation, clustering, filtering and assignment of mutations to clusters (Fig. 1).

#### 2.2.1 Cell frequency estimation

We consider two types of molecular mechanisms that convert a locus into its mutated state: copy number variation (CNV) inducing events and single nucleotide variation (SNV) inducing events. We assume that a normal state is defined by a total ploidy of two and B allele ploidy below two, whereas a mutated state has an increased fraction of B alleles. The conditions defining these states for each locus *l* are formulated below:
(1)
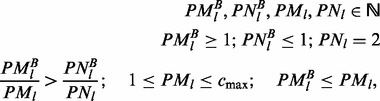


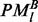
 and 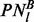 denote the ploidy of the B allele in each cell type: mutated cells and normal cells, respectively. The value of 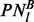 is one if *l* has a germline variant, zero otherwise. 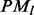 and 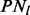 are the total ploidy of mutated cells and normal cells. 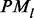 is required to be between one and *c_max_*, that is, we exclude solutions for which the maximum number of amplicons per cell exceeds the user-defined constant *c_max_*. The choice of *c_max_* should depend on genomic depth of coverage and on the fraction of the genome sequenced: the higher the quality and abundance of data, the higher *c_ma__x_*.

For each locus *l,* the equations below contrast the measured total ploidy (2) and B allele ploidy (3) of all cells in the sample to the sum of cell type-specific ploidies:
(2)
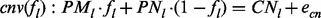

(3)
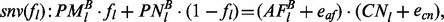

Where 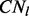 and 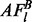 denote the copy number and B allele frequency measured for *l* and 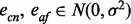 is normally distributed noise that reflects the uncertainties in copy number and allele frequency measurements, respectively. We calculate the fraction 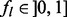 of mutated cells with respect to locus *l* from the aforementioned equations for all ploidy combinations (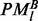, 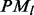) that satisfy 1. For each solution, we calculate an error-term 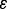 as the deviation of the measured from the expected allele frequency and copy number:
(4)
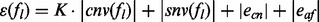

where 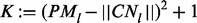 is a penalty factor in favor of ploidies that are close to the measured copy number. The probability 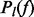 that the mutation at locus *l* is present in a fraction *f* of cells is obtained by fitting a Gaussian mixture model on 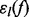 (Fig. 1A). As the equation system given by (2) and (3) is underdetermined, we obtain multiple solutions for the same locus (Supplementary Fig. S1). For example, in a copy neutral region, the allele frequency of a heterozygous mutation present in 100% of the cells should be the same as that of a homozygous mutation present in only 50% of the cells. The ambiguity of the solutions depends on the measured allele frequency: low or high allele frequencies have a constricting effect on 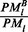, increasing the kurtosis of 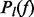. In contrast, moderate allele frequencies result in more uniformly distributed solutions.

#### 2.2.2 Clustering

Next we find overrepresented cell frequencies using a two-step clustering procedure. Based on the assumption that passenger mutations occur within a cell before the driver event that initiates the expansion, each clonal expansion should be marked by multiple mutations.

Thus SNVs and CNVs that took place in a cell before a clonal expansion should be present in a similar fraction of cells and leave a similar trace in the subsequent clonal expansion. The aim is to find common peaks in the distribution of 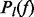 for multiple mutated loci *l*. In the first step, mutations with similar 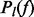 are grouped together by hierarchical cluster analysis of the probability distributions 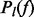 using the Kullback–Leibler divergence as a distance measure (Fig. 1B). The joint probability distribution of cell frequencies is computed for each cluster *C* as follows:

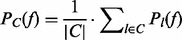

The cell frequency 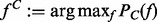 at each cluster-maxima denotes the size of the SP that harbors the clustered mutations. In the second step, each cluster *C* is extended by members with similar distributions in an interval around the cluster-maxima 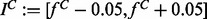 (highlighted in red in Fig. 1C).

#### 2.2.3 Filtering

Next, we keep only those clusters for which 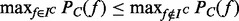 is rejected at a user-defined *P*-value (underlying statistical test: paired *t*-test). A rejection indicates that mutations clustered in *C* are all present in the same fraction of cells 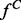 (Fig. 1C). The number of remaining clusters denotes the number of predicted clonal expansions. Tumor purity is inferred as the size of the largest SP.

#### 2.2.4 Assignment of SNVs to clusters

Finally, we assign each locus *l* to 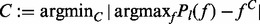 (Fig. 1D). The mutated loci assigned to each cluster represent the genetic profile of each predicted SP.

### 2.3 Validation and performance

We tested the performance of EXPANDS on a simulated and two real datasets: 118 GBM exome sequencing samples from TCGA (Supplementary Table S2) and one whole genome sequenced breast cancer. Somatic point mutations and LOH for each GBM sample were obtained by applying Mutect (Cibulskis *et al.*, 2013) on the tumor-derived BAM file and the patient-matched normal BAM file. Copy-number segments were calculated using an approach similar to ExomeCNV (Sathirapongsasuti *et al.*, 2011). SNVs outlying autosomes or that cannot be explained by an SP present in 10% or more of the sample (i.e. AF × CN < 0.1) were excluded.

#### 2.3.1 Simulation

To investigate how the abundance of mutations in a sample and the noise in their measurement affect the accuracy of SP predictions, a simulated dataset was generated, consisting of *n = 350* samples. In each sample, we simulate 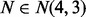, 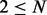 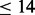 clonal expansions, each marked by a variable number *t* of mutations, 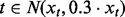. Each mutation was simulated as a triplet 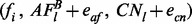, where 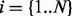, 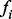 is the size of 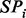 and 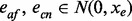 are normally distributed noise in allele frequency measurements and in the estimation of local copy number, respectively. We choose 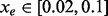 to reflect distinct ranges of genomic depth of coverage (as described in Supplementary Information: the effect of genomic depth of coverage on allele frequency noise). Allele frequency and copy number were calculated according to Equations 1–3 from 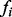 and 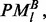 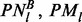, simulated as Poisson distributions (see Supplementary Table S1). Mutations that are fixed in the tumor cell population are present in the same fraction of cells, regardless whether they emerged during one or during multiple clonal expansions (see Supplementary Fig. S2). To account for the fact that we cannot see past the last fixation event, we further require that the first clonal expansion harbors the majority of mutations among all simulated expansions and that it gives rise to an SP present in >50% of the tumor (i.e. simulated samples have >50% tumor purity).

**Fig. 2.**
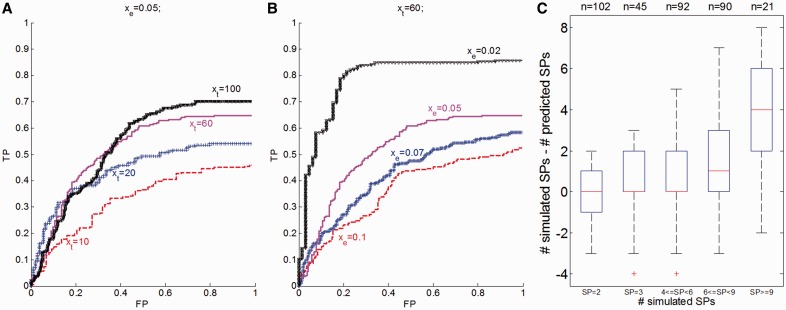
EXPANDS—validation experiment on simulated dataset. SP prediction accuracy is shown for variable simulation parameters. A total of 1621 clonal expansions were simulated among 350 tumors. (**A** and **B**) Receiver Operating Curve (ROC) of SP size prediction accuracy. (A) Each clonal expansion was represented by varying number of mutations 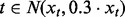 at a constant noise rate x_e_ = 0.05. (B) A variable noise term 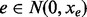 was added to the copy number and allele frequency of simulated mutations at a constant number of mutations per clonal expansion x_t_ = 60. (**C**) Deviation between simulated and predicted number of SPs is shown for various numbers of simulated SPs for all 350 tumors

We predict the SP sizes 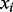 from the simulated allele frequencies and copy numbers using EXPANDS and obtain a *P*-value for each predicted SP, reflecting the confidence with which the SP has been detected. We vary the upper threshold for the *P*-value below which we accept an SP and denote predictions for which the deviation between true and predicted SP size: 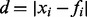 is below 0.03 as true predictions. Figure 2A and B shows the results of the validation experiment for varying 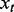 and 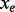. As expected, we observe an increase in the precision of EXPANDS with increasing 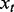 and decreasing 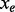. The numbers of simulated and predicted SPs are mostly consistent (Fig. 2C). However, EXPANDS underestimates the true number of distinct SPs in samples with many coexisting SPs. Because SPs are detected by their relative size in the tumor bulk, we expect more false-negative (FN) predictions in samples with many SPs, as SPs are more likely to have similar sizes. Furthermore, we observe an enrichment of SP size 50% among FNs. This effect is a direct consequence of the decreased kurtosis of cell frequency probability distributions derived from moderate allele frequencies. Both sources for FNs are illustrated in Supplementary Figure S3.

We conclude that EXPANDS predicts the number and size of SPs with 50–80% accuracy given enough genomic depth of coverage and fraction of the genome sequenced. On data of lower depth and breadth of coverage, the number of SPs predicted by EXPANDS should not be considered absolute values, but can provide a qualitative comparison among samples.

#### 2.3.2 Dependence of EXPANDS prediction sensitivity on mutation count

The results of the simulation experiment described previously indicate that the prediction accuracy of EXPANDS increases with the number of mutations that mark an SP (Fig. 2A), which in turn depends on the genomic breadth of coverage. We estimated the prediction accuracy of EXPANDS on mutations detected in a hypermutated ER-positive breast cancer genome—PD4120a (Nik-Zainal *et al.*, 2012). The whole genome of this breast cancer has been sequenced at 188-fold coverage, providing high-quality data to investigate how representative different areas of the genome are of coexisting SPs. We applied EXPANDS to 7175 mutations scattered across the exome and additional surrounding regions (total 300 MB). The predicted SPs were compared with SPs inferred by Nik-Zainal *et al.* from mutations found in the whole genome. The sizes of the SPs inferred by Nik-Zainal *et al.* were multiplied by tumor purity (0.7), to make them comparable with our predictions.

We found considerable overlap between our predictions and the observations by Nik-Zainal *et al.* For example, EXPANDS inferred the same pattern of mutations and copy number alteration in the two dominating SPs that were detected in the founder clone inferred by Nik–Zainal. The details of the overlap are described in Supplementary Figure S4. In addition to the four mutation clusters identified by Nik-Zainal *et al.*, EXPANDS detected yet another two SPs present in 27 and 19% of the sample, respectively, both marked by increasing genomic instability. These observations confirm substantial subclonal copy number variations that occur rather late in tumor development, as reported by Nik-Zainal *et al.* Importantly, the analysis shows that ∼10% of the mutations detected in the hypermutated breast cancer genome are sufficient to predict the SP composition of this tumor at a resolution comparable with that obtained from the whole genome.

**Fig. 3.**
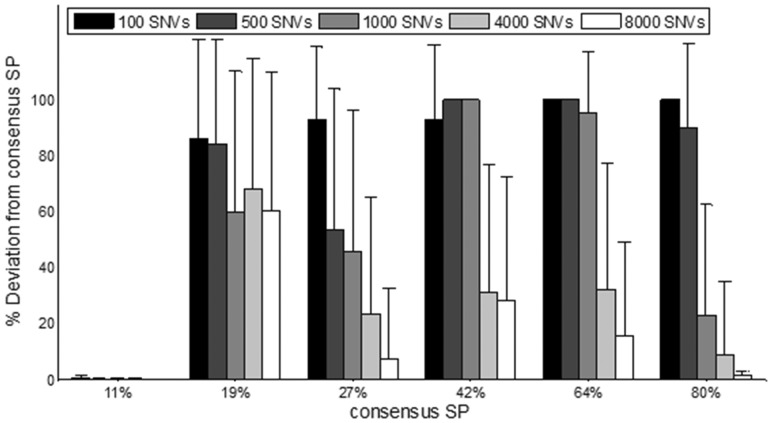
EXPANDS prediction accuracy depends on mutation abundance. Six consensus SPs were identified based on the allele frequency and copy number of 7175 mutations detected within a hypermutated ER-positive breast cancer genome (*x*-axis). The size of the consensus SPs was compared with the size of SPs predicted based on mutations found in non-overlapping regions of variable length. Mean deviation of predicted SP size from each consensus SP size (*y*-axis) decreases with increasing number of SNVs

To estimate how breadth of coverage affects the prediction sensitivity of EXPANDS, we approximate the dependence of prediction sensitivity on mutation abundance. We applied EXPANDS on mutations found in 123 non-overlapping genomic regions of variable length and compared the predicted SPs with the consensus SPs described previously. We calculated the deviation of the predicted SP size from the size of the consensus SP (see Fig. 3). A deviation of 100% marked the absence of the corresponding consensus SP from the predictions. As suggested by the simulated dataset, SPs present in ∼50% of the sample are more difficult to detect and require a higher number of marker mutations. The 19% SP was marked by a lower fraction of mutations such that a higher fraction of the genome was required to observe enough mutations that are specific to this SP. In contrast, SP-specific SNVs for the 11% SP were overrepresented among all SNVs due to its tetraploid genome (Supplementary Fig. S4). The high fraction of the genome mutated in this SP allowed for its detection even at low mutation count.

Our results indicate that mutations found in a region twice as large as the cancer exome are sufficient to identify at least 50% of the SPs with 90% probability. We conclude that EXPANDS can identify genetically distinct SPs that coexist in a tumor sample based on only a fraction of the mutations present in that sample. However, mutation rate and size distribution of SPs in the tumor impact the sensitivity of the method. By modeling cellular mutation frequencies as probability distributions, EXPANDS postpones having to decide on fixed cellular mutation frequencies and can thereby resolve the clonal composition of this complex genome that harbors many copy number variations.

#### 2.3.3 Robustness and specificity of subpopulation predictions

We tested the robustness of SP size predictions among independent sets of non-overlapping SNVs detected in the same exome sample. As this approach required splitting the SNVs detected in one sample, we required that at least 290 SNVs (somatic mutations and LOH) have been detected in total in that sample. This criterion applied to 53 of the 118 GBM exome sequencing samples from TCGA (Supplementary Table S2). For each sample *k*, we split the detected somatic SNVs and LOH, in two non-overlapping sets of similar size (*SOM1 + LOH1* and *SOM2 + LOH2*). For each SNV set (number of SNVs in each set: 128–3897), we predict the SPs for sample k:

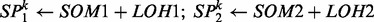

We choose a *P*-value of 0.005 to accept a SP. No SPs could be detected in nine cases at this *P*-value. We then compare the consistency between predictions within the same sample 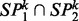 to that between predictions of pairwise distinct samples: 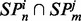, where 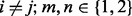. To exclude run-specific effects as the cause of robust predictions, we required that sample *i* and *j* were processed on the same plate and that a similar number of SNVs (max. deviation: 50 SNVs) were detected in each member of the sample pair. Two SPs were considered overlapping if their size was <0.03 apart. The fraction of overlapping SPs among the matching pairs (mean = 0.59) was significantly increased (*t*-test: *P* = 5.3E-12) compared with the random pairs (mean = 0.23), indicating that two mutually exclusive sets of SNVs from the same sample reveal similar SPs (Supplementary Fig. S5).

Here we used the predicted SP size to validate the robustness of SP predictions. The size of a SP correlates almost exactly with its SNV content as shown in Supplementary Figure S6, and can therefore be used as a measure of SP identity. We conclude that EXPANDS can identify tumor-specific clonal expansions and the size of the resulting SPs in the tumor bulk in a robust manner.

#### 2.3.4 Tumor purity predictions

Next we compared tumor purity predictions by EXPANDS with those performed by ABSOLUTE (Carter *et al.*, 2012) and to histological purity estimates.

We applied EXPANDS to allele frequency and copy number measurements obtained from exome sequences. We assumed that the largest SP predicted by EXPANDS is a product of the first or multiple early clonal expansion(s). Therefore, we expected the size of the largest SP to predict the percentage of tumor cells in the sample, i.e. tumor purity. In addition, we predicted tumor purity using ABSOLUTE from copy number measurements obtained from SNP6 array data. Both approaches were applied to identical areal samples of 66 GBM, which are a subset of the samples introduced in Section 2.3.

**Fig. 4.**
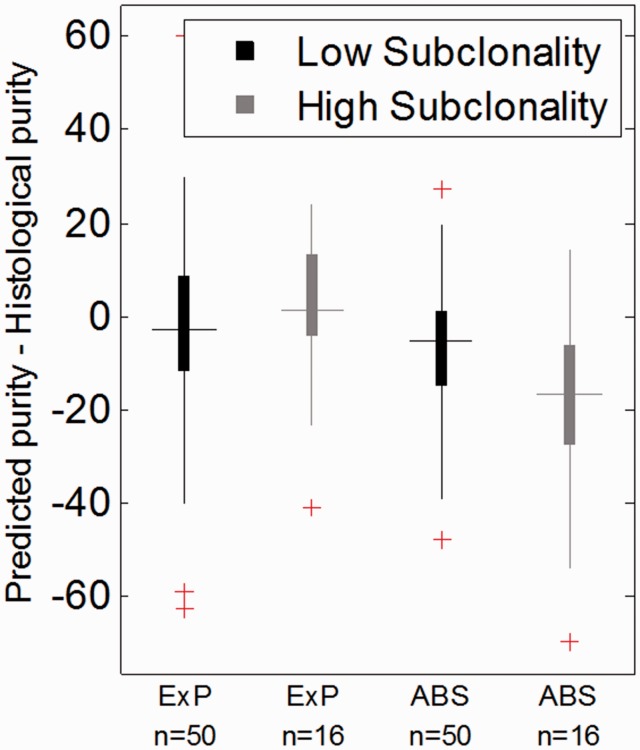
Comparison of tumor purity prediction approaches. The fraction of tumor cells in each of 66 GBM samples were predicted by EXPANDS (ExP) and ABSOLUTE (ABS). The deviation between predicted tumor purity and histological purity estimates was compared between samples of low and high subclonality. Note that ABSOLUTE and EXPANDS performed similarly on samples of low to moderate subclonality. EXPANDS provided estimates of tumor purity that were closer to histological purity estimates in samples of high subclonality than ABSOLUTE (*t*-test: *P* = 9.3E-4)

We found that tumor purities predicted by ABSOLUTE and EXPANDS were largely consistent (Fig. 4), with a median deviation of 0.11. One possible explanation for the deviation between the predictions by the two approaches is that high numbers of subclonal SNVs and CNVs interfere with purity estimations. It is noteworthy that ABSOLUTE detects subclonal mutations by allowing for deviations in copy number from the discrete levels. However, this strategy supports only a moderate fraction of subclonal events (Carter *et al.*, 2012). To determine whether high subclonality impedes tumor purity predictions by ABSOLUTE, we quantified tumor heterogeneity. To do this, we took the number and size of SP predictions made by EXPANDS and computed the Shannon index for each sample. The Shannon index is a common measure of species diversity in ecology and has recently been adopted to quantify diversity in tumor samples (Merlo *et al.*, 2010).

For samples with a low to moderate Shannon index (lower 0.75 quantile), EXPANDS and ABSOLUTE performed similarly with respect to histology purity predictions. Notably, EXPANDS tumor purity predictions were closer to histological purity estimates than ABSOLUTE in samples with high intra-tumoral heterogeneity (upper 0.25 quantile) (Fig. 4). Thus, we conclude that ABSOLUTE underestimates purity for tumors with high subclonality and that EXPANDS is the preferred approach for tumor purity predictions in highly heterogeneous samples.

## 3 RESULTS

### 3.1 Predicting GBM subpopulation dynamics

EXPANDS complements tumor purity estimations with predictions of the size and clonal composition of SPs and the identification of mutations that mark these SPs within a single tumor.

To predict the SP composition of GBM at diagnosis, we applied EXPANDS to somatic mutation and LOH detected in the tumors of 108 GBM patients available at TCGA. Our results indicate that each GBM at the time of surgery consists of between 1 and 16 SPs (median = 7). Not only did the number of SPs differ, but also the genetic profiles of individual SPs were highly diverse among patients. The predicted size of the SPs ranged between 10 and 100% of the total cells in the sample, with a median of 42 SNVs per SP (Supplementary Table S2). The data show feasibility that EXPANDS can be applied to a sizeable patient group to predict the clonal evolution individually for each tumor patient.

For 10 of the 108 patients, exome data from patient-matched primary and recurrent GBM were available (Supplementary Fig. S7) and were analyzed for SP changes on recurrence, in the context of clinical information.

**Fig. 5.**
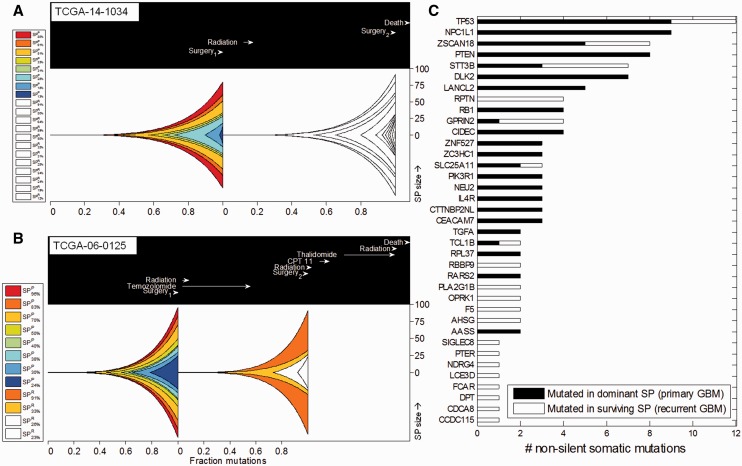
Genetic changes in primary and recurrent GBM SPs. The predicted clonal composition of matched primary and recurrent GBM is shown for two patients: (**A**) TCGA-14-1034 and (**B**) TCGA-06-0125. Genetically unique clones emerge as a consequence of accumulating beneficial mutations and expand into SPs (represented by different colors). The lower *x*-axis shows the relative timing of clonal expansions by indicating the fraction of mutations that have accumulated in the entire tumor before the onset of each expansion. The upper box indicates timing of clinical events relative to the time from tumor initiation to first surgery (set to 237 days—the mean time between the first and the second surgery among the 10 matched patients). The *y*-axis indicates the percent (%) representation of each SP in the sequenced tumor bulk at the time of the first and second surgery. EXPANDS infers the presence of multiple SPs that coexist in the primary tumor at first surgery. After the first surgery, (sometimes followed by radiation, chemotherapy), the tumor recurs and EXPANDS infers the evolution of the recurrent tumor SPs. Each SP in the recurrent tumor is colored based on its predicted ancestor in the primary tumor. New SPs that were absent or undetectable in the primary GBM and emerged only on recurrence remain white. Note that the SP composition and dynamics in the two patients are different. Both patients start out with eight SPs in the primary tumors. The recurrent tumor of TCGA-14-1034 harbors 12 coexisting SPs that share little similarity to the primary SPs. In contrast the recurrent tumor of TCGA-06-0125 has only four SPs, two of which could be assigned to primary SPs. (**C**) Candidate driver genes mutated in fittest GBM SPs. Significantly mutated genes in selected SPs of 69 primary and 10 recurrent GBM samples as predicted by MutSig. The *x*-axis indicates the number of non-silent somatic mutations detected in the genes listed on the *y*-axis. Genes were mutated either in the dominant SPs of primary tumors (black) or in the surviving SPs of recurrent tumors (white)

In patient TCGA-14-1034, 8 SPs were detected in the primary tumor and 12 SPs in the recurrent tumor. Strikingly, dominant SPs detected in the recurrent tumor shared no significant proportion of SNVs with SPs from the primary tumor (Fig. 5A).

In contrast, in patient TCGA-06-0125, eight SPs were detected in the primary tumor and four in the recurrent tumor. Two of these SPs shared a significant fraction of SNVs between primary and recurrent tumor (Fig. 5B) suggesting that these recurrent SPs descend from primary SPs that have presumably survived treatment. Genes mutated in the treatment-surviving SPs include those with brain-specific tissue expression: NPBWR2, NEFH, GLRA3, CHAT and NDRG4 and those with a function in cell growth, differentiation, mitotic cycle and oncogenic transformation, such as PTPN11—a member of the protein tyrosine phosphatase family (Pruitt *et al.*, 2012).

In contrast, the other two SPs in this recurrent tumor could not be assigned to any SP in the primary GBM. These new SPs were marked by 30 non-silent mutations, including missense mutations in MAP2K3 (a member of the MAP kinase kinase family) and PLXNC1 (a member of the plexin family). These two cases thus showed different SP composition dynamics with recurrence. Analyses of eight additional cases further confirmed that SP compositions invariably change with recurrence and that SP dynamics during recurrence are highly individualized. One scenario observed was that none of the SPs detected in the primary tumor was dominant on recurrence (TCGA-14-1034; TCGA-06-0152; TCGA-06-0171). In the second scenario, some SPs survive whereby in 4 of 10 cases one of the dominant SPs in the primary tumor persisted as dominant SP in the recurrent tumor (TCGA-06-0190, TCGA-06-0211, TCGA-06-0221 and TCGA-06-0125). In the remaining cases, smaller SPs of the primary tumor became dominant on recurrence (TCGA-19-4065; TCGA-06-0210; TCGA-14-0736) (Fig. 5 and Supplementary Fig. S8).

Notably, for patients who received standard treatment (radiation and temozolomide) between the first and second surgery, we observed a by trend elevated numbers of mutations in the recurrent as compared with the primary tumor (*t*-test: *P* = 0.12, data not shown). Moreover, we observed that temozolomide treatment before recurrence shows a tendency to co-occur with a decreased number of SPs in the recurrent tumor as compared with the primary GBM. In contrast, patients who did not receive chemotherapy and radiation therapy had an elevated number of SPs in the recurrent tumor as compared with the primary GBM (*t*-test: *P* = 0.08, Supplementary Fig. S9).

Taken together based on data from a small sample set, we provide evidence that tumor recurrence significantly changes the SP composition of tumors and is highly individualized. Data from this limited sample set can be used in conjunction with clinical information to formulate a hypothesis on the effects of temozolomide treatment on intra-tumoral heterogeneity and test it on a large dataset using EXPANDS.

### 3.2 Identification of candidate genes affecting subpopulation fitness

Next, we aimed to identify genes that when mutated provide a substantial selective advantage to SPs in primary and recurrent GBM. To this end, we first identified the SPs present in at least 60% of the sample (SP size 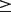
0.6) as the dominant SPs in the primary tumors, further denoted as SP^D^. We rationalized that SNVs that mark these SP^D^ are of interest as they might contribute to the successful growth of SP^D^s and thus to overall tumor growth and recurrence. We then applied MutSig (Lawrence *et al.*, 2013) on 5173 somatic SNVs assigned to 136 SP^D^ from 69 primary GBM patients whereby we identified genes that were mutated in SP^D^ more often than expected by chance. Not surprisingly, the top 20 significant hits identified by MutSig were enriched for genes previously known to be involved in gliomagenesis: TP53, PTEN, PIK3R1 and TGFA (Pruitt *et al.*, 2012). The enrichment of these gene alterations in SP^D^ suggests that they are highly prevalent in individual tumors and further corroborate their role as GBM driver mutations. A detailed view of the intra-tumoral prevalence of known GBM driver mutations is provided in Supplementary Figure S10. In addition to known GBM drivers, the top hits list contained genes previously not associated with GBM, such as DLK2, CEACAM7 and RPL37 (Fig. 5C and Supplementary Table S3)—novel candidate genes to be tested for their role in tumorigenesis.

Next, we aimed to identify those genes that when mutated provide SPs with a selective growth advantage on treatment and are thus important for recurrence. SPs found in the recurrent tumor, could either have survived treatment and stem from an ancestral SP in the primary tumor or could have emerged *de novo* perhaps due to treatment. We identified the ancestral SP for each SP in the recurrent tumor by taking into account size and shared mutation frequency: We hypothesize that two SPs that share many of their mutations are likely to be closely related clones. Second, SPs that were small in the primary tumors and became large (dominant) on recurrence potentially carry a genetic signature that is relevant for recurrence (Supplementary Fig. S2). Based on this hypothesis, we assume that the ancestral SP is the smallest SP in the primary tumor that shares a minimum of 10% of the mutations assigned to the SP in the recurrent tumor. SPs found in the recurrent tumor for which we could identify an ancestral SP are further denoted SP^R^.

We found 887 SNVs assigned to 26 SP^R^ detected in eight recurrent GBM patients. Among the 20 most significantly mutated genes were regulators of transforming growth factor beta signaling (RBBP9 and DPT) and regulators of AKT signaling (TCL1B) (Pruitt *et al.*, 2012) as well as other genes that have not been tested specifically for their role in GBM recurrence (Supplementary Table S3).

Taken together, these findings show that EXPANDS can guide the analysis of SP dynamics on recurrence and can be used in combination with clinical data to correlate treatment effects with tumor heterogeneity. EXPANDS generates mutational profiles of dominant SPs in primary and recurrent tumors that can be used to find novel candidate genes with potential functions in GBM growth and recurrence, respectively.

## 4 DISCUSSION

### 

#### 4.1.1 Tumor heterogeneity and purity predictions by EXPANDS

We present EXPANDS, a method to systematically identify and characterize tumor heterogeneity and clonal SP dynamics. To our knowledge, it is the first automated method that predicts intra-tumoral genetic heterogeneity from sequencing data of moderate coverage by assessing clonal composition of tumors using allele fractions of single nucleotide variants and copy number changes.

EXPANDS presents several useful features that provide an improvement over existing approaches: it can be applied to single tumor samples and is amenable to analyze differences in tumor heterogeneity within large datasets. Our approach detects differences in the number, distribution and content of SPs among GBM and led us to conclude that individual and independent tumor profiles need to be generated to properly describe intra-tumoral heterogeneity in GBM. It will be interesting to apply EXPANDS to other tumor types to determine the extent and pattern of heterogeneity. Our method is informative for tumor purity estimation, even for samples of high subclonality, where EXPANDS purity predictions were closer to histological estimates than tumor purity predictions by ABSOLUTE.

Another feature of EXPANDS is that it predicts SPs that can share a subset of their mutations/CNVs and thus can be nested within each other. In contrast, Tolliver *et al.* describes tumor mixture separation from gene expression or aCGH data but requires convex combinations of SP sizes—that is, the approach detects SPs with mutually exclusive properties (Tolliver *et al.*, 2010). This is achieved based on the assumptions that genes/probes with similar patterns across multiple subpopulations are eliminated in a principal component analysis step. In addition, the number of SPs is required as an input. Finally, the method by Tolliver *et al.* requires multiple tumor-samples (distinct patients) as an input, whereas EXPANDS predicts SPs independently, from sequencing data obtained from individual patients.

The validation of SP content, i.e. the accuracy with which SNVs are assigned to individual SPs, is not feasible in the absence of geographical sampling. However, EXPANDS assigns SNVs to SPs based on the fraction of cells that harbor the SNV. The SNVs assigned to a SP in turn determine the size of the SP. Therefore, SP size is highly correlated to SP content and can be used as a measure of SP similarity (Supplementary Fig. S6). In future experiments, SP sizes estimated by EXPANDS will be validated by other methods such as fluorescence *in situ* hybridization.

#### 4.1.2 Clinical information and EXPANDS

The genetic and phenotypic cellular heterogeneity is a hallmark of many cancers that obscures the contribution of individual cancer cells to tumor progression and therapy resistance. Studies in Barrett's esophagus (Merlo *et al.*, 2010) and head and neck squamous cell carcinoma (Mroz *et al.*, 2013) showed that high genetic heterogeneity estimated from geographical studies predicts poor prognosis. This intra-tumor heterogeneity can explain the incomplete response of tumors to therapeutic intervention and tumor recurrence. The number of coexisting SPs predicted by EXPANDS and their size provide a quantitative measure of tumor heterogeneity and can be translated into common measures of diversity, such as the Simpsons and Shannon indices. Using EXPANDS data on a small number of cases, we observed a tendency of primary GBM treated with temozolomide to recur with a reduced number of SPs. Temozolomide treatment has previously been shown to provide a survival benefit for GBM patients. A larger dataset is needed to confirm or refute that temozolomide consistently decreases SP heterogeneity on recurrence. If confirmed it will be interesting to determine whether reduced SP heterogeneity is predictive of a survival benefit for treatments in general.

#### 4.1.3 Candidate gene predictions derived from EXPANDS data

EXPANDS predicts what mutations mark coexisting SPs at the time of tumor detection and can thereby help guide therapeutic choices such that multiple SPs can be targeted and eliminated simultaneously. We have identified a list of genes significantly mutated in dominant SPs in the primary tumors. These include genes previously implicated in GBM such as PTEN and TP53. Importantly, the list also includes novel genes without a previously demonstrated function in gliomagenesis. These genes may be tested by molecular functional experiments for their role in tumor formation.

In addition, our analyses provided a list of genes mutated in SPs that survived in the recurrent tumors. In future experiments, our method will be applied on a large number of samples to identify SPs that survived therapy and recurrent mutations within these SPs as candidates with a testable role in promoting recurrence. In combination with clinical information, EXPANDS will be useful to test for genetic determinants of therapy response and could be of predictive value.

### 4.2 The role of CNV in the detection of subpopulation size

EXPANDS uses the copy number and the B allele frequency of mutated loci to infer the frequency of cells that harbor these mutations. The advantage of clustering the inferred cell frequencies over allele frequency clustering lies in the dependence of allele frequencies on copy number. Copy number changes within any cell in the sequenced sample affect the measured allele frequency. As an example, we consider a diploid locus *l_2_* and a haploid locus *l_1_*, where each locus has exactly one mutated allele 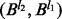 and both mutations co-occur in an SP of size 0.6 (60% of the cells have both, 40% have neither mutation). Owing to different underlying copy numbers, the expected allele frequency of 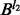 is 0.3 and that of 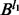 is 0.6, despite their common SP-origin. We conclude that copy number has to be included to accurately estimate the fraction of cells that harbor specific mutations, especially in cancer-genomes with high genetic instability, where CNVs are abundant.

Allele frequency distributions show a similar pattern across tumor samples in general. A perfectly pure tumor sample, with no normal cell contamination and one homogeneous tumor cell population is expected to have a few homozygous mutations with a peak at 1.0 and heterozygous mutations visible at a peak around 0.5. However, because tumor samples are often contaminated with normal cells, both peaks are typically shifted to the left, on average around 0.7 and 0.35. In addition, peaks below 0.2 indicate the presence of subclonal mutations (Supplementary Fig. S11). SNVs within LOH are visible as one peak above 0.8. The increased allele frequency of SNVs within LOH regions is caused by the presence of the germline variant in at least one copy of all cells.

In contrast to allele frequency distributions, the distributions of cell frequencies are distinct among tumor samples and increase the resolution on the SPs present in that sample. The adjustment of allele frequencies by copy numbers allows the inclusion of germline variants within LOH regions in the SP size predictions. As prediction accuracy increases with the number of passenger mutations that occur in a cell before its expansion, the abundance of LOH makes these events a valuable resource in the SP size prediction, especially in tumor types with low numbers of somatic point mutations.

### 4.3 Limitations and outlook

An underlying assumption of our model is that SP-specific mutations are present in a fraction of cells that is specific to the SP in which these mutations reside. As a consequence, two or more distinct SPs that coexist in the same tumor at equal proportions will be identified as just one SP. The sensitivity with which EXPANDS distinguishes individual SPs, that is, how much two SPs have to differ in their size for them to be recognized as two distinct SPs, depends on the noise in copy number and allele frequency measures, the number of SNVs that mark each of these SPs and the size of the SPs itself. In the case of NGS data, this translates to a dependence on coverage, base- and mapping qualities as well as consistent experimental conditions for tumor and control samples.

A limitation of this current study is the sample size used in the analysis of matched primary and recurrent GBM. Providing answers to the question of how therapy affects SP composition on recurrence and what genetic changes provide SPs from the recurrent tumor with the ability to survive therapy will require a larger set of trios and a larger proportion of the cancer genomes sequenced (e.g. whole genome sequencing) to increase the resolution on SP content.

EXPANDS allows for quantification of tumor purity and of heterogeneity via Shannon index, Simpsons index and genetic divergence (Merlo *et al.*, 2010). In the future, EXPANDS will be applied to a larger patient group for which sequencing data are available to determine tumor purity and SP composition. EXPANDS will be useful in the context of clinical information to reveal possible associations between the extent of intra-tumoral heterogeneity, the genetic profile of individual SPs and clinical outcome. Studies will be extended to investigate possible relationships between therapeutic intervention and specific patterns of SP dynamics in GBM and other cancer types.

Moreover, longitudinal studies of SPs predicted by EXPANDS can help determine whether SP-specific gene mutations pre-existed in the primary tumor or were acquired *de novo* during recurrence and thus are expected to provide new insights into the mechanisms for recurrence.

## 5 CONCLUSIONS

We present an approach that predicts the number of SPs that coexist in a tumor, the size of the SPs in the tumor bulk, the mutations that mark each SP and the extent of normal cell contamination. This information will be useful to complement geographical sampling, especially in the case of intermixed tumor cell populations. EXPANDS can be applied on SNVs and CNVs derived from sequencing data of various depth and breadth of coverage including exome and whole genome sequencing data and is unique in that it takes an unbiased approach to provide an unprecedented extent of individualized predictions. It can be applied to all types of cancer for which NGS data are available, to address crucial questions about tumor heterogeneity and recurrence mechanisms.

## Supplementary Material

Supplementary Data
