# Supplementary material for: EXPANDS: expanding ploidy and allele frequency on nested subpopulations
Source: Bioinformatics. 2013 Oct 30;30(1):50–60. doi: 10.1093/bioinformatics/btt622 (PMC3866558; doi:10.1093/bioinformatics/btt622)
Supplement: Supplementary Data [file supp_30_1_50__index.html]

EXPANDS: Expanding Ploidy and Allele Frequency on Nested Subpopulations — EXPANDS: expanding ploidy and allele frequency on nested subpopulations — EXPANDS: expanding ploidy and allele frequency on nested subpopulations — Supplementary Data 

# EXPANDS: expanding ploidy and allele frequency on nested subpopulations

## Supplementary Data

files

**Files in this Data Supplement:**

- Supplementary Data - pdf file
