# Supplementary material for: EXPANDS: expanding ploidy and allele frequency on nested subpopulations
Source: Bioinformatics. 2013 Oct 30;30(1):50–60. doi: 10.1093/bioinformatics/btt622 (PMC3866558; doi:10.1093/bioinformatics/btt622)
Supplement: Supplementary Data [file supp_btt622_Supplementary-Andor_et_al.pdf]

EXPANDS: Expanding Ploidy and Allele Frequency on Nested Subpopulations

Supplementary Information

Noemi Andor, Julie Harness, Hans Werner Mewes and

Claudia Petritsch

The effect of genomic depth of coverage on allele frequency noise: Let L be the locus of interest and c be the total count of reads overlapping L. Further let $AF^B = \frac{b}{c}$ and $X^B = \frac{x}{c}$ be the true and observed allele frequency of the B-allele at locus L respectively, such that $x = b$ reads matching the B-allele would lead to the exact estimation of the correct allele frequency. Then x, the measured count of reads matching the B-allele, can be modeled as a Poisson distribution: $x \sim P(\mathrm{b})$ with mean b and variance b. It follows that the variance in measured allele frequency:

$$Var(X^B) = \frac{1}{c^2} \bullet Var(\mathrm{x}) = \frac{1}{c^2} \bullet b$$

Consequently, the measured allele frequency of a SNV present in 0.8 of the DNA in the sequenced sample would have a variance of 0.04 at 20 fold coverage, while at 100 fold coverage the variance would decrease to 0.008. The estimation above takes into account only the stochastic noise and can be considered a lower threshold of the true noise, which is further influenced by sequencing errors and mapping errors. We conclude that the modeled noise-range $\mathrm{e} \in \mathrm{N}(0, \mathrm{x}_e)$, with $x_e \in [0.02, 0.1]$ is realistic and applies to depth of coverage dependent sequencing projects.

Supplementary Table S1: (Relevant for Fig 2). Simulation parameters. U – uniform distribution; P – Poisson distribution; N – normal distribution.

Parameter Name	Parameter	Distribution
Cell-frequency	f	$U(0,1)$
Error in allele frequency- and copy number measurement	e_{af}, e_{cn}	$N(0, x_e)$
Number of mutations per subpopulation	t	$N(x_t, 0.3{*}x_t)$
Total ploidy in mutated cells at mutated locus	PM_l	$1+\frac{P(1)}{2}$
Ploidy of B-allele in mutated cells	PM_l^B	$1+\frac{P(1)}{2}$
Ploidy of B-allele in non-mutated cells	PN_l^B	$\frac{1}{1+P(0.7)}$

Supplementary Table S2. Clonal expansions detected among 118 GBM samples (108 primary GBM – green; 10 recurrent GBM - red). For each sample, the predicted number of SPs is shown along with the number of LOH/somatic SNVs (including intronic and silent mutations) and tumor-purity estimations for the sequenced tumor bulk.

Sample ID	# SNVs (LOH and somatic)	# SPs	Tumor-purity (largest SP)
TCGA-02-0003-01A-01D-1490-08.4	943	9	0.89
TCGA-02-0033-01A-01D-1490-08.4	269	4	0.27
TCGA-02-0047-01A-01D-1490-08.4	305	6	0.63
TCGA-02-0055-01A-01D-1490-08.4	398	8	0.74
TCGA-06-0125-01A-01D-1490-08.6	203	8	0.96
TCGA-06-0125-02A-11D-2280-08.1	251	5	0.91
TCGA-06-0132-01A-02D-1491-08.5	168	4	0.43
TCGA-06-0151-01A-01D-1491-08.5	217	8	0.80
TCGA-06-0152-01A-02D-1492-08.5	477	8	0.79
TCGA-06-0152-02A-01D-2280-08.1	350	9	0.79
TCGA-06-0154-01A-03D-1491-08.5	293	6	0.90
TCGA-06-0157-01A-01D-1491-08.5	213	9	0.94
TCGA-06-0158-01A-01D-1491-08.5	305	7	0.86
TCGA-06-0165-01A-01D-1491-08.5	182	1	0.27
TCGA-06-0166-01A-01D-1491-08.5	238	5	0.94
TCGA-06-0167-01A-01D-1491-08.5	205	3	0.22
TCGA-06-0168-01A-01D-1491-08.5	276	8	0.79
TCGA-06-0171-01A-02D-1491-08.5	264	7	0.81
TCGA-06-0171-02A-11D-2280-08.1	350	4	0.65
TCGA-06-0173-01A-01D-1491-08.5	458	7	0.85
TCGA-06-0174-01A-01D-1491-08.5	1983	10	0.94
TCGA-06-0178-01A-01D-1491-08.5	277	9	0.84
TCGA-06-0184-01A-01D-1491-08.5	297	6	0.57
TCGA-06-0185-01A-01D-1491-08.5	579	14	0.90
TCGA-06-0187-01A-01D-1491-08.8	351	6	0.60
TCGA-06-0188-01A-01D-1491-08.5	288	10	0.76
TCGA-06-0189-01A-01D-1491-08.5	147	6	0.40
TCGA-06-0190-01A-01D-1491-08.5	1661	8	0.53
TCGA-06-0190-02A-01D-2280-08.1	344	7	0.86
TCGA-06-0192-01B-01D-1492-08.4	287	6	0.62
TCGA-06-0195-01B-01D-1491-08.5	1207	9	1.00
TCGA-06-0209-01A-01D-1491-08.5	341	6	0.49
TCGA-06-0210-01B-01D-1491-08.5	246	6	0.62
TCGA-06-0210-02A-01D-2280-08.1	281	11	1.00
TCGA-06-0211-01B-01D-1491-08.5	277	6	0.59
TCGA-06-0211-02A-02D-2280-08.1	281	8	1.00
TCGA-06-0213-01A-01D-1491-08.5	260	5	0.77
TCGA-06-0214-01A-02D-1491-08.5	314	7	0.42
TCGA-06-0216-01B-01D-1492-08.4	1447	14	1.00

TCGA-06-0219-01A-01D-1491-08.5	258	9	0.62
TCGA-06-0221-01A-01D-1491-08.5	203	5	0.44
TCGA-06-0221-02A-11D-2280-08.1	403	5	0.97
TCGA-06-0237-01A-02D-1491-08.5	1989	15	0.91
TCGA-06-0240-01A-03D-1491-08.6	97	6	0.58
TCGA-06-0241-01A-02D-1491-08.5	237	5	0.36
TCGA-06-0644-01A-02D-1492-08.5	217	6	0.82
TCGA-06-0645-01A-01D-1492-08.5	247	8	0.49
TCGA-06-0646-01A-01D-1492-08.5	253	7	1.00
TCGA-06-0648-01A-01D-1492-08.5	284	7	0.89
TCGA-06-0649-01B-01D-1492-08.9	257	6	0.84
TCGA-06-0686-01A-01D-1492-08.5	284	8	0.76
TCGA-06-0744-01A-01D-1492-08.4	796	5	0.98
TCGA-06-0745-01A-01D-1492-08.4	1255	5	1.00
TCGA-06-0747-01A-01D-1492-08.4	2108	11	0.99
TCGA-06-0749-01A-01D-1492-08.4	271	4	0.35
TCGA-06-0750-01A-01D-1492-08.4	304	6	0.77
TCGA-06-0876-01A-01D-1492-08.4	572	10	0.93
TCGA-06-0877-01A-01D-1492-08.4	365	9	0.82
TCGA-06-0878-01A-01D-1492-08.4	512	9	0.79
TCGA-06-0879-01A-01D-1492-08.4	394	9	0.91
TCGA-06-0881-01A-02D-1492-08.4	208	5	0.72
TCGA-06-0882-01A-01D-1492-08.4	176	6	0.63
TCGA-06-1806-01A-02D-1845-08.2	66	NA	NA
TCGA-06-6388-01A-12D-1845-08.2	96	6	0.83
TCGA-06-6693-01A-11D-1845-08.2	1575	10	0.89
TCGA-06-6694-01A-12D-1845-08.2	182	8	0.68
TCGA-06-6695-01A-11D-1845-08.2	100	NA	NA
TCGA-06-6698-01A-11D-1845-08.2	7012	16	1.00
TCGA-06-6699-01A-11D-1845-08.2	121	NA	NA
TCGA-06-6700-01A-12D-1845-08.2	104	4	0.79
TCGA-06-6701-01A-11D-1845-08.2	89	NA	NA
TCGA-12-0615-01A-01D-1492-08.5	325	14	0.98
TCGA-12-0616-01A-01D-1492-08.5	207	7	0.99
TCGA-12-0618-01A-01D-1492-08.5	290	7	0.88
TCGA-12-0619-01A-01D-1492-08.5	312	9	0.94
TCGA-12-0688-01A-02D-1492-08.4	411	10	1.00
TCGA-12-0692-01A-01D-1492-08.4	204	15	1.00
TCGA-14-0736-01A-01D-2280-08.1	178	7	0.93
TCGA-14-0736-02A-01D-2280-08.1	123	6	0.53
TCGA-14-0740-01B-01D-1845-08.2	154	9	0.93
TCGA-14-0787-01A-01D-1492-08.4	1386	8	0.91
TCGA-14-0789-01A-01D-1492-08.4	320	9	0.93
TCGA-14-0813-01A-01D-1492-08.4	475	9	0.58
TCGA-14-0817-01A-01D-1492-08.4	1027	13	0.80

TCGA-14-0862-01B-01D-1845-08.2	102	NA	NA
TCGA-14-0871-01A-01D-1492-08.5	3479	13	1.00
TCGA-14-1034-01A-01D-1492-08.7	303	8	0.80
TCGA-14-1034-02B-01D-2280-08.1	1758	13	0.91
TCGA-14-1043-01B-11D-1845-08.2	76	5	0.68
TCGA-14-1395-01B-11D-1845-08.2	137	7	0.79
TCGA-14-1450-01B-01D-1845-08.2	107	4	0.70
TCGA-14-1823-01A-01D-1494-08.4	240	7	0.83
TCGA-14-1825-01A-01D-1494-08.4	766	6	1.00
TCGA-14-1829-01A-01D-1494-08.4	329	5	1.00
TCGA-15-0742-01A-01D-1492-08.4	1570	13	0.96
TCGA-16-0846-01A-01D-1492-08.4	618	10	0.88
TCGA-16-0848-01A-01D-1492-08.4	608	10	0.94
TCGA-16-0861-01A-01D-1492-08.4	1015	4	0.94
TCGA-19-4065-01A-01D-2280-08.1	146	5	0.79
TCGA-19-4065-02A-11D-2280-08.1	265	6	0.74
TCGA-19-5953-01B-12D-1845-08.2	146	6	0.56
TCGA-26-6173-01A-11D-1845-08.2	114	6	0.65
TCGA-26-6174-01A-21D-1845-08.2	163	8	0.81
TCGA-27-1830-01A-01D-1494-08.4	309	5	0.79
TCGA-27-1833-01A-01D-1494-08.4	772	4	0.90
TCGA-27-1834-01A-01D-1494-08.4	1032	6	0.81
TCGA-28-5211-01C-11D-1845-08.2	37	NA	NA
TCGA-32-2498-01A-01D-1353-08.3	214	7	0.65
TCGA-41-6646-01A-11D-1845-08.2	254	8	0.89
TCGA-74-6573-01A-12D-1845-08.2	142	5	0.97
TCGA-74-6575-01A-11D-1845-08.2	181	7	0.72
TCGA-74-6577-01A-11D-1845-08.2	109	5	0.78
TCGA-74-6578-01A-11D-1845-08.2	134	9	0.94
TCGA-74-6581-01A-11D-1845-08.1	684	8	1.00
TCGA-76-6280-01A-21D-1845-08.2	146	7	0.62
TCGA-76-6283-01A-11D-1845-08.2	125	6	0.74
TCGA-76-6286-01A-11D-1845-08.2	890	10	0.92
TCGA-76-6656-01A-11D-1845-08.2	193	10	1.00

Supplementary Table S3. (Relevant for Fig. 5 C). Candidate driver genes mutated in fittest GBM SPs detected in 69 primary and 10 recurrent GBM samples. Shown are gene function (Franceschini *et al.*, 2013), the p-value and the total number of nonsilent somatic mutations detected by Mutsig in genes that are significantly mutated either in the dominant SPs of the primary tumors or in the surviving SPs of the recurrent tumors.

		dominant primary GBM SPs		surviving recurrent GBM SPs	
**gene**	**function**	**p**	**n_nonsilent**	**p**	**n_nonsilent**
PTEN	Phosphatase and tensin homolog; Tumor suppressor. A	0.00	8	1.00	0
TP53	Tumor protein p53; Acts as a tumor suppressor in many tumor types; induces growth arrest or apoptosis depending on the physiological circumstances and cell type.	1.00E-05	9	2.70E-04	3
DLK2	Epidermal growth factor-like protein 9.	3.00E-05	7	1.00	0
RPL37	Ribosomal protein L37; Binds to the 23S rRNA.	1.00E-04	2	1.00	0
CEACAM7	carcinoembryonic antigen-related cell adhesion molecule 7 .	4.00E-04	3	1.00	0
NPC1L1	NPC1 (Niemann-Pick disease, type C1, gene)-like 1; Play a major role in cholesterol homeostasis.	1.15E-03	9	1.00	0
NEU2	Sialidase 2 (cytosolic sialidase); Hydrolyzes sialylated compounds .	1.40E-03	3	1.00	0
TGFA	Transforming growth factor, alpha; TGF alpha is a mitogenic polypeptide that promotes anchorage-independent cell proliferation in soft agar.	1.72E-03	2	1.00	0
LANCL2	LanC lantibiotic synthetase component C-like 2 (bacterial); Necessary for abscisic acid (ABA) binding on the cell membrane and activation of the ABA signaling pathway in granulocytes	2.60E-03	5	1.00	0
PIK3R1	Phosphoinositide-3-kinase, regulatory subunit 1 (alpha); Binds to activated (phosphorylated) protein-Tyr kinases, through its SH2 domain	5.04E-03	3	1.00	0
ZNF527	Zinc finger protein 527; May be involved in transcriptional regulation.	5.18E-03	3	1.00	0
CIDEC	Cell death-inducing DFFA-like effector c; Induces apoptosis .	5.57E-03	4	1.00	0
ZSCAN18	Zinc finger and SCAN domain containing 18; May be involved in	5.68E-03	5	4.30E-04	3

	transcriptional regulation				
SLC25A11	Solute carrier family 25 (mitochondrial carrier; oxoglutarate carrier), member 11.	6.42E-03	2	7.97E-03	1
AASS	Aminoadipate-semialdehyde synthase; Bifunctional enzyme that catalyzes the first two steps in lysine degradation.	6.59E-03	2	1.00	0
ZC3HC1	Zinc finger, C3HC-type containing 1; Essential component of an SCF-type E3 ligase complex, SCF(NIPA), a complex that controls mitotic entry by mediating ubiquitination and subsequent degradation of cyclin B1 (CCNB1).	7.27E-03	3	1.00	0
CTTNBP2NL	CTTNBP2 N-terminal like	7.54E-03	3	1.00	0
IL4R	Interleukin 4 receptor; Receptor for both interleukin 4 and interleukin 13. Couples to the JAK1/2/3-STAT6 pathway. The IL4 response is involved in promoting Th2 differentiation.	8.68E-03	3	1.00	0
RARS2	Arginyl-tRNA synthetase 2, mitochondrial	1.02E-02	2	1.00	0
RB1	Retinoblastoma 1; Key regulator of entry into cell division that acts as a tumor suppressor.	1.04E-02	4	1.00E+00	0
TCL1B	T-cell leukemia/lymphoma 1B; Enhances the phosphorylation and activation of AKT1 and AKT2.	1.54E-02	1	5.22E-03	1
STT3B	STT3, subunit of the oligosaccharyltransferase complex, homolog B.	0.11	3	1.30E-04	4
GPRIN2	G protein regulated inducer of neurite outgrowth 2.	0.21	1	3.00E-05	3
OPRK1	Opioid receptor, kappa 1; Inhibits neurotransmitter release by reducing calcium ion currents and increasing potassium ion conductance.	1.00	0	3.00E-05	2
RPTN	Repetin; Involved in the cornified cell envelope formation. Multifunctional epidermal matrix protein.	1.00	0	8.10E-04	4
F5	Coagulation factor V (proaccelerin, labile factor); Central regulator of hemostasis.	1.00	0	1.89E-03	2
PLA2G1B	Phospholipase A2, group IB (pancreas); PA2 catalyzes the calcium-dependent hydrolysis of the 2- acyl groups in 3-sn-	1.00	0	3.23E-03	2

	phosphoglycerides.				
RBBP9	Retinoblastoma binding protein 9; May play a role in the transformation process due to its capacity to confer resistance to the growth-inhibitory effects of TGF-beta1 through interaction with retinoblastoma and the subsequent displacement of E2F-1.	1.00	0	3.27E-03	2
LCE3D	Late cornified envelope 3D; Precursors of the cornified envelope of the stratum corneum.	1.00	0	4.03E-03	1
AHSG	Alpha-2-HS-glycoprotein; Promotes endocytosis, possesses opsonic properties and influences the mineral phase of bone. Shows affinity for calcium and barium ions	1.00	0	5.49E-03	2
CDCA8	Cell division cycle associated 8; Component of the chromosomal passenger complex (CPC), a complex that acts as a key regulator of mitosis.	1.00	0	6.35E-03	1
CCDC115	Coiled-coil domain containing 115	1.00	0	7.38E-03	1
DPT	Dermatopontin; Seems to mediate adhesion by cell surface integrin binding. Enhances TGFB1 activity. Inhibits cell proliferation.	1.00	0	7.49E-03	1
SIGLEC8	Sialic acid binding Ig-like lectin 8; Putative adhesion molecule that mediates sialic-acid dependent binding to cells.	1.00	0	7.64E-03	1
PTER	phosphotriesterase related.	1.00	0	7.67E-03	1
FCAR	Fc fragment of IgA, receptor for; Binds to the Fc region of immunoglobulins alpha. Mediates several functions including cytokine production.	1.00	0	8.94E-03	1
NDRG4	NDRG family member 4; May play a role in the early postnatal development and function of neuronal cells.	1.00	0	9.13E-03	1

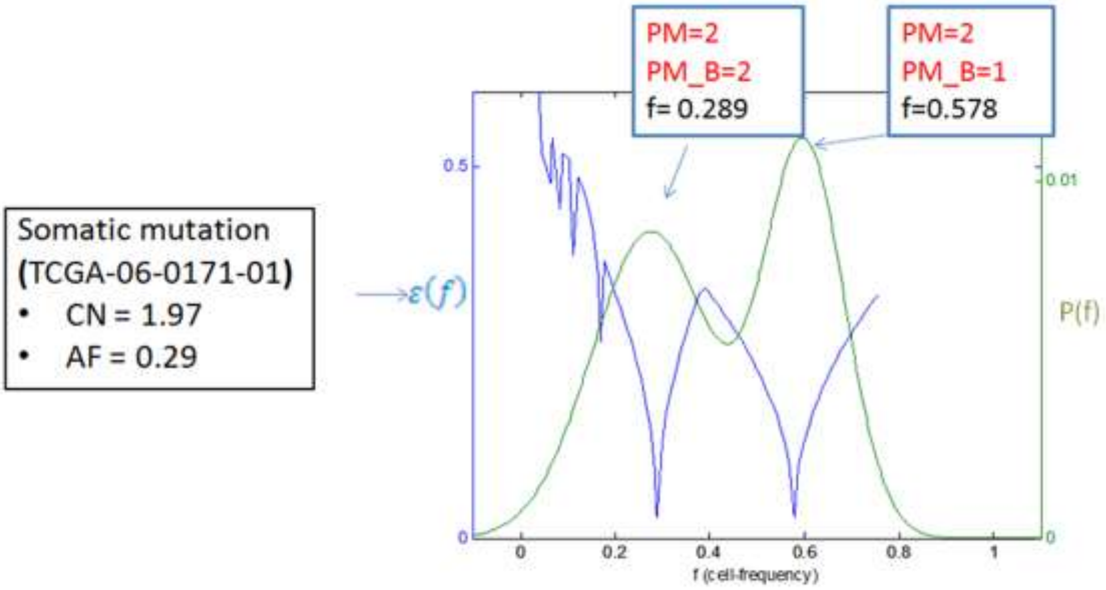

Supplementary Figure S1 (Relevant for Fig. 1A). **Probability distribution of cell-frequencies for a somatic mutation in TCGA-06-0171**. The deviation $\varepsilon(f)$ reflects how well a cell-frequency can explain the observed copy number (CN) and allele-frequency (AF). The blue curve shows the deviation ε from a perfect fit while the green line is the function fitted on $\varepsilon(f)$: $P(f)$ - reflecting the probability that this mutation is present in a fraction f of cells. This is a representative ambiguous case in which the observations can be explained by different cellular frequencies: either a homozygous mutation present in ~29% of the cells or a heterozygous mutation present in ~58% of the cells.

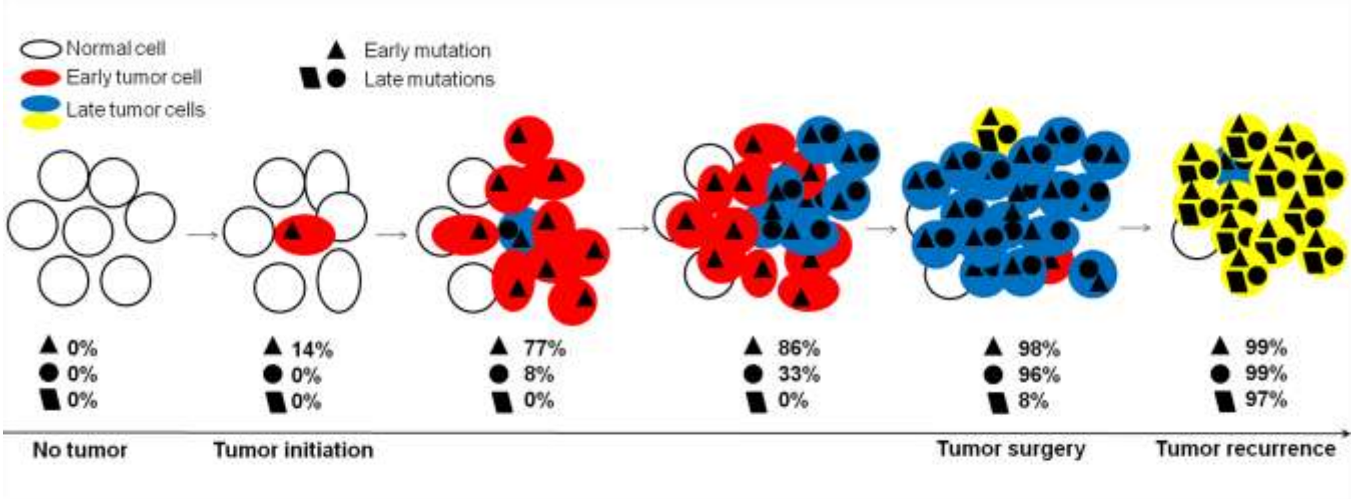

Supplementary Figure S2: Expected outcome of EXPANDS subpopulation predictions on a simulated case. Tissue composition is given for an area of constant size at multiple time points. Mutations accumulate in tumor cells continuously from the time of tumor initiation to surgery (arrow) and cause clonal expansions. Cells are shown as circles. Mutations found in early tumor cells are a subset of mutations found in late tumor cells. The frequencies of early and late mutations among all cells at a relative time point are indicated. At the time of tumor-surgery two somatic mutations, represented by triangles and circles, are present at similar cellular frequencies in the tumor bulk. Consequently EXPANDS will predict that both mutations belong to the same subpopulation although they emerged during distinct clonal expansions. The third mutation represented by a square, would be assigned to a second clonal expansion. Upon tumor-recurrence, the yellow subpopulation has become abundant, suggesting a potential role of the mutation represented by a square, for therapy resistance.

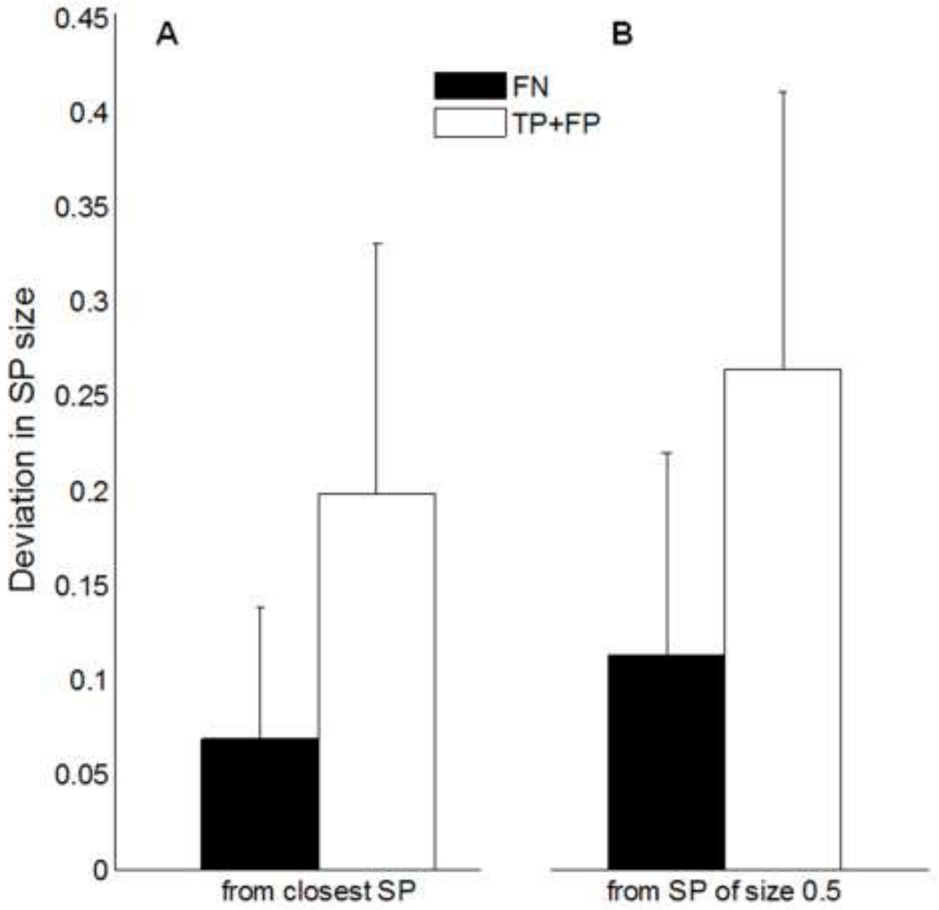

Supplementary Figure S3: **Causes for false negative (FN) predictions by EXPANDS on simulated dataset**. A) As our simulations did not require SPs to have distinct sizes, about 18% of SPs simulated within one tumor overlap in their size with one other SP simulated within the same tumor (within 0.05 distance). As SPs are identified by their relative size in the tumor bulk, two SPs that have very similar size will be identified as just one single SP (FNs). B) SPs present in ~50% of the sample are represented by a wider range of allele frequencies and ploidities. As a result, the kurtosis at ~50% SP size is lower, making SPs of this size more difficult to detect.

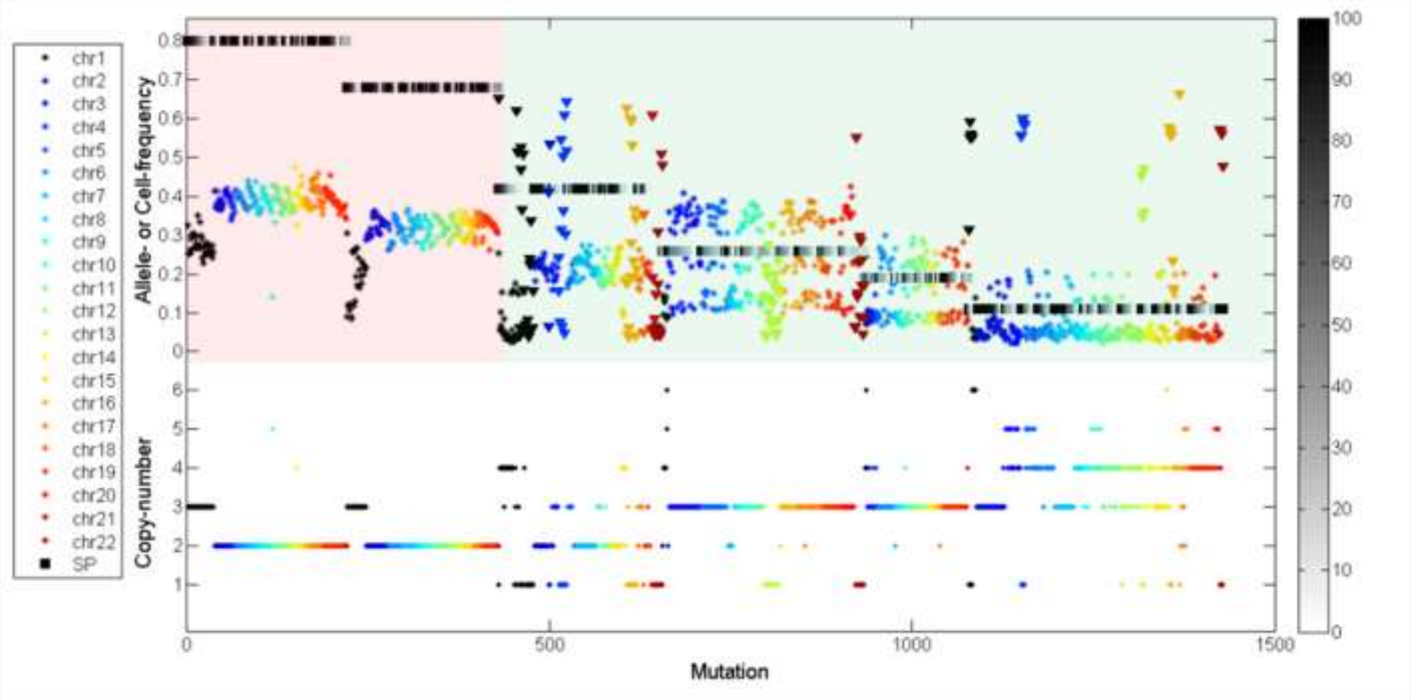

Supplementary Figure S4: (Relevant for Fig. 3) Coexistent subpopulations determined by EXPANDS in an ER-positive breast cancer genome (Nik-Zainal *et al.*, 2012). Six subpopulations (SPs) were identified based on the allele-frequency and copy number of 7175 mutations detected within the cancer-genome. SPs were present in 80%, 64%, 42%, 27%, 19% and 10% of the sample (y-axis). For each of the 1428 exonic mutations (x-axis) we show: - the SP to which the mutation has been assigned (squares), - the ploidy of the locus in that SP and - the allele frequency of the mutation. Allele frequencies and ploidities are colored based on the chromosome on which the mutation is located (stars – somatic SNVs, triangles - LOH). SPs are colored based on the confidence with which the mutation has been assigned to the SP (black - highest, white - lowest). SPs present in a large portion of the sample (>50%) are the result of early clonal expansions (upper left corner: red background). Early SPs have mutations in TP53, GATA3, MLL3 and NCOR1 and are hypo-diploid, with single-copy-gain on chr1 being the earliest major copy number change. The very same mutations and copy number alteration were found in the founder clone detected by Nik-Zainal et al. The third clonal expansion detected by EXPANDS gave rise to a SP present in 42% of the sample and was similar to the 47% SP detected by Nik-Zainal et al. in that it had only few somatic point mutations, and was dominated by single-copy losses and LOH. Chromosomal instability is an ongoing process in later stages (upper right corner: green background) with the last clonal expansion giving rise to a tetraploid SP present in 10% of the sample. In addition to the four mutation-clusters identified by Nik-Zainal et al., EXPANDS detected yet another two SPs present in 27% and 19% of the sample respectively, both marked by increasing genomic instability.

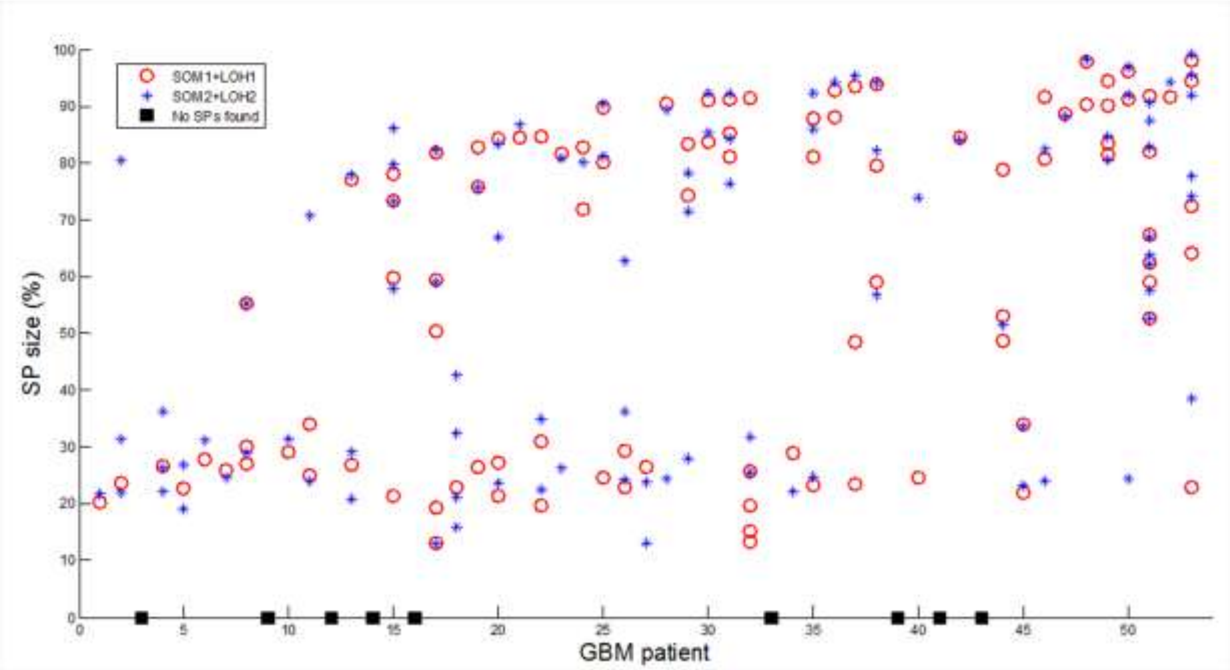

Supplementary Figure S5: Prediction accuracy of SP size. Prevalence of tumor cell SPs inferred from exome-capture sequencing data. SP-size predicted on 53 GBM exomes from TCGA. Each symbol represents a SP predicted to exist in the given percentage (y-axis) of the corresponding sample (x-axis). For each sample the detected mutations were separated into two non-overlapping sets of similar size. SPs were predicted independently on each set (circles and stars).

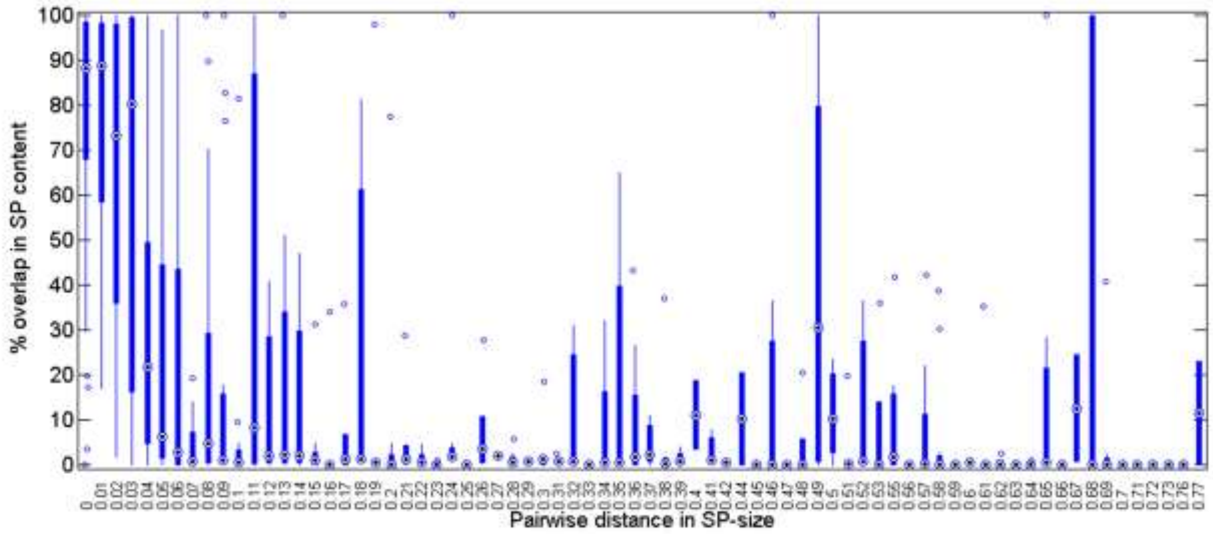

Supplementary Figure S6: Correlation between SP size and SP content. EXPANDS was used to predict coexisting SPs in 53 GBM: once using all SNVs detected in the exonic regions of the tumors and once using only 50% of the SNVs. The two predictions are not independent from each other as the list of SNVs used as an input overlap. The overlap is required to address SP-similarity as a function of SP content. The size difference of all pairwise SPs predicted for the same tumor (x-axis) using the two different SNV sets, is plotted against the percentage of SNVs that overlap (with respect to the smaller set). Notably, the SP content overlap dramatically decreased at SP size distances above 0.03. Occasionally, heterozygous and homozygous mutations are confounded, leading to a median SP content overlap of ~30% around SP size distance 0.49. The high percentage of overlapping SNVs between SPs of similar size indicates that both, SP size and SP content can be used as a measure of SP identity.

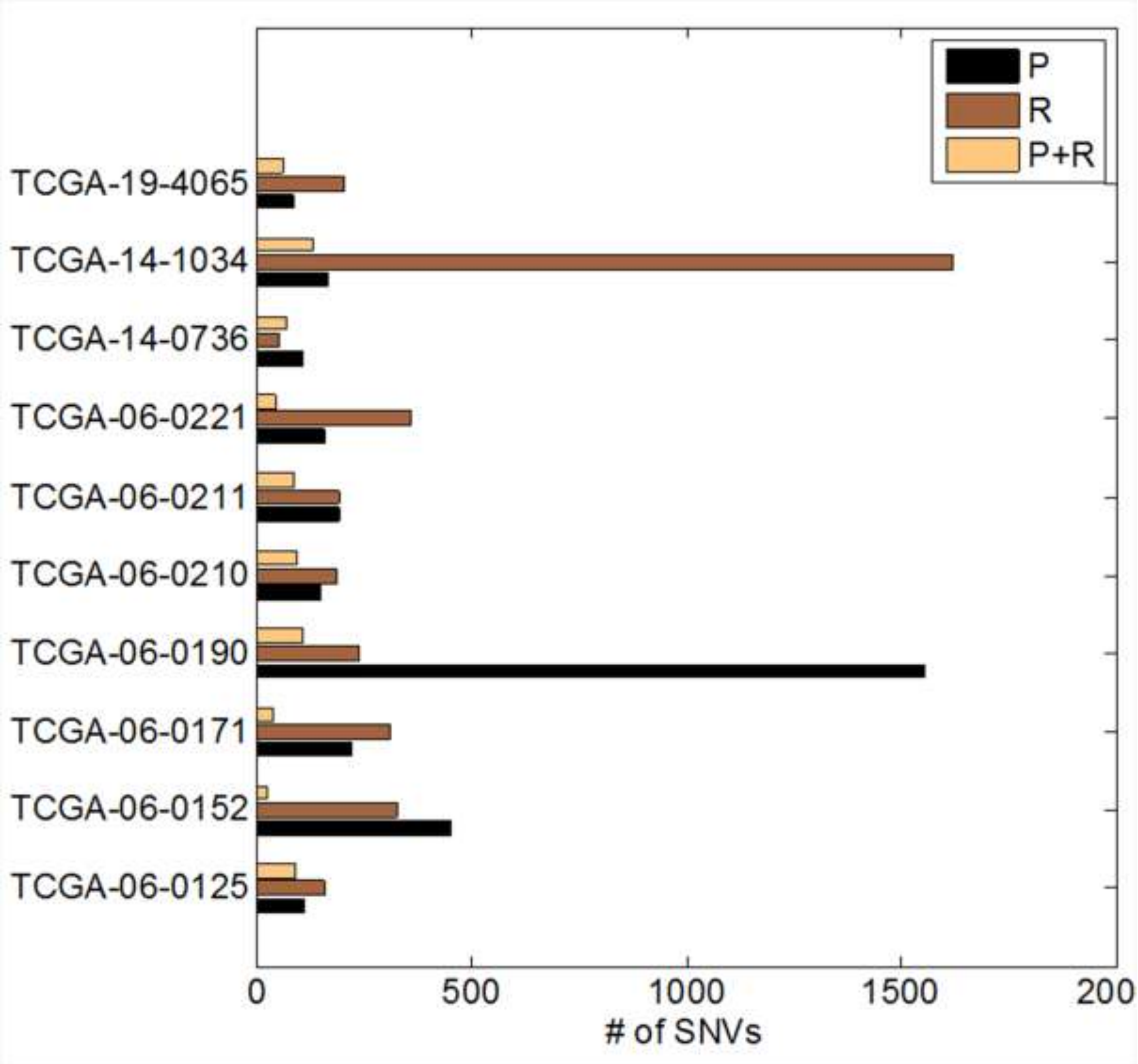

Supplementary Figure S7: Somatic mutations and LOH detected in 10 matched primary and recurrent GBM. Numbers of somatic mutations and LOH unique to primary (P) or recurrent (R) GBM samples are displayed together with SNVs that are common between matched primary and recurrent samples (P+R). Relevant for Fig. 5 in main text.

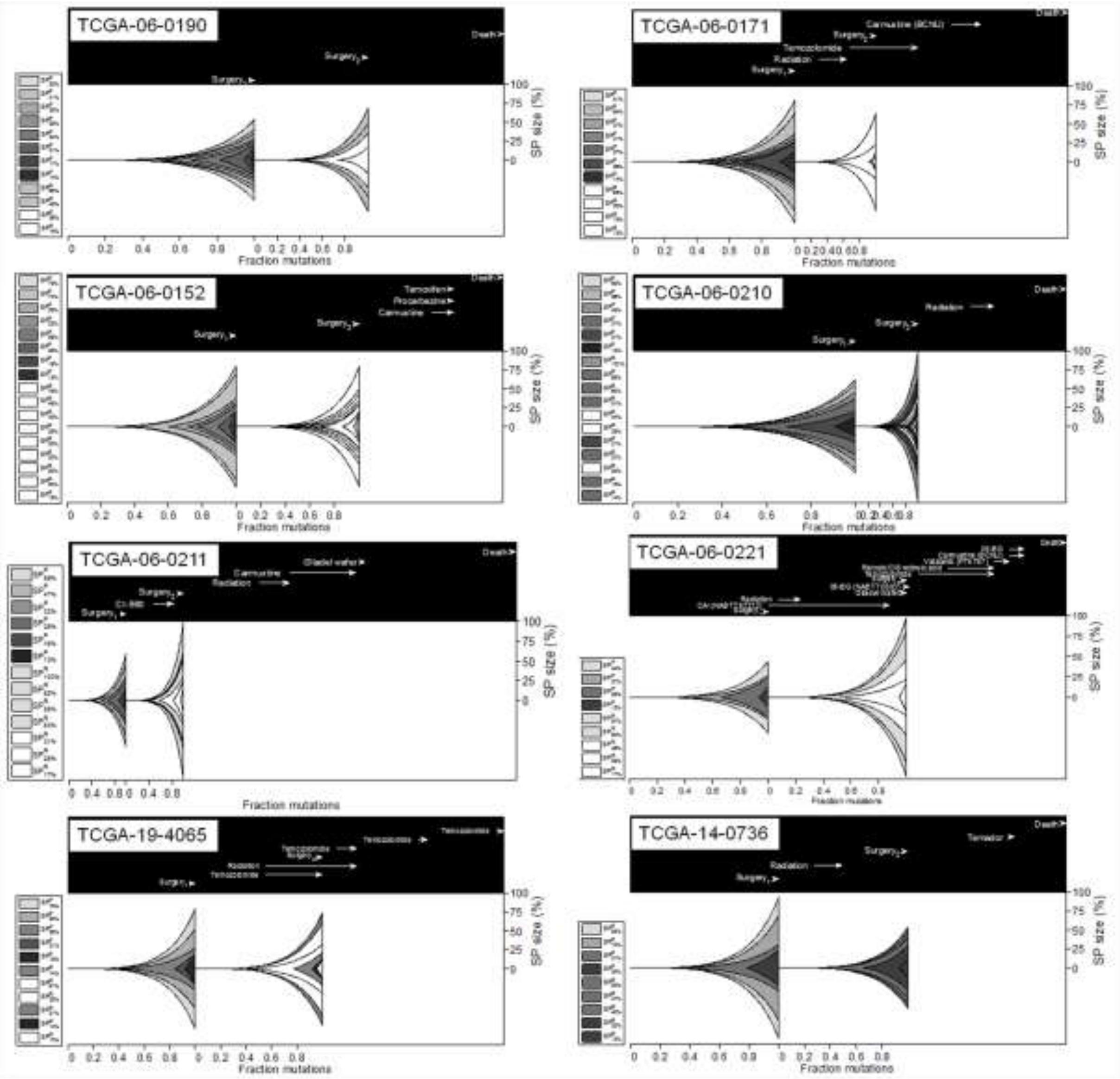

Supplementary Figure S8: (Relevant for Fig. 5) **Clonal expansions and evolving SPs in primary and recurrent GBM**. The predicted clonal evolution of matched primary and recurrent GBM is shown for eight patients. Genetically unique clones emerge as a consequence of accumulating beneficial mutations and expand into SPs (represented by different colors). The lower x-axis shows the relative timing of clonal expansions by indicating the fraction of mutations that have accumulated in the tumor before the onset of each expansion. The upper box indicates the relative timing of clinical events: the time from tumor initiation to first surgery is set to 237 days (the mean time between the first and the second surgery among the ten matched patients). The y-axis indicates the percent (%) representation of each SP in the sequenced tumor bulk at the time of the first and second surgery. EXPANDS infers the presence of multiple SPs that coexist in the primary tumor at first surgery. After the first surgery, radiation and chemotherapy the tumor recurs and EXPANDS infers the evolution of the recurrent tumor SPs. Each SP in the recurrent tumor is colored based on its predicted ancestor in the primary tumor. New SPs that were absent or undetectable in the primary GBM and emerged only upon recurrence are colored white.

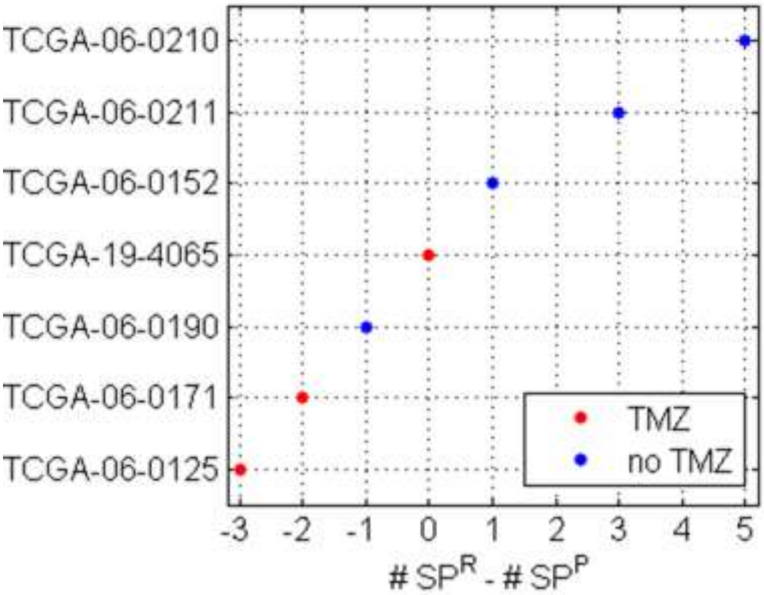

Supplementary Figure S9: Effect of Temozolomide on the number of subpopulations in recurrent GBM. Difference between number of subpopulations (SPs) observed in recurrent (SP^R) and primary (SP^P) GBM is compared among patients who received adjuvant chemotherapy with Temozolomide (*n=3*) and untreated patients (*n=4*). In Temozolomide-treated patients the recurrent tumors have a decreased number of SPs as compared to the primary GBM. In contrast, in untreated GBM the number of SPs in the recurrent tumor is higher in ¾ cases (75%) than the number of SPs in the primary GBM (ttest; p-value 0.082).

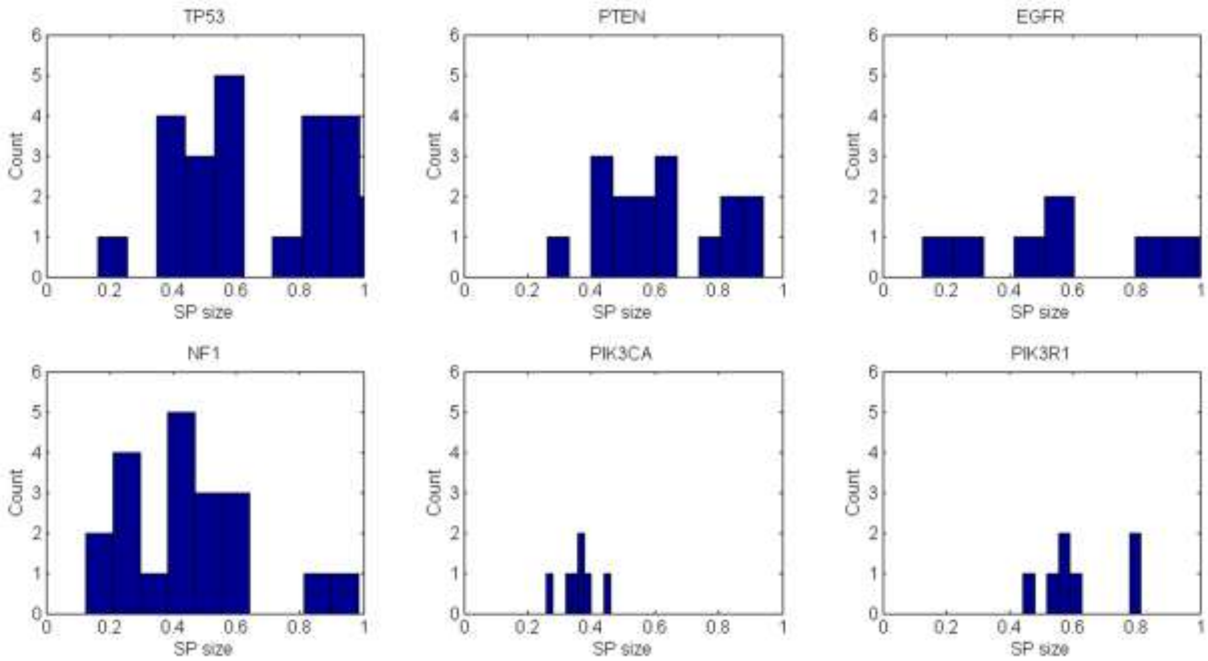

Supplementary Figure S10: Intra-tumoral prevalence of mutations within GBM driver genes. Shown is the SP size distribution assigned to all mutations (including silent and intronic) detected in TP53, PTEN, EGFR, NF1, PIK3CA and PIK3R1 among 70 GBM samples. A negative skew in the SP-size distribution indicates that the corresponding gene is often mutated in the majority of the sequenced cells, while a positive skew suggests that only a subset of tumor cells harbor the mutations.

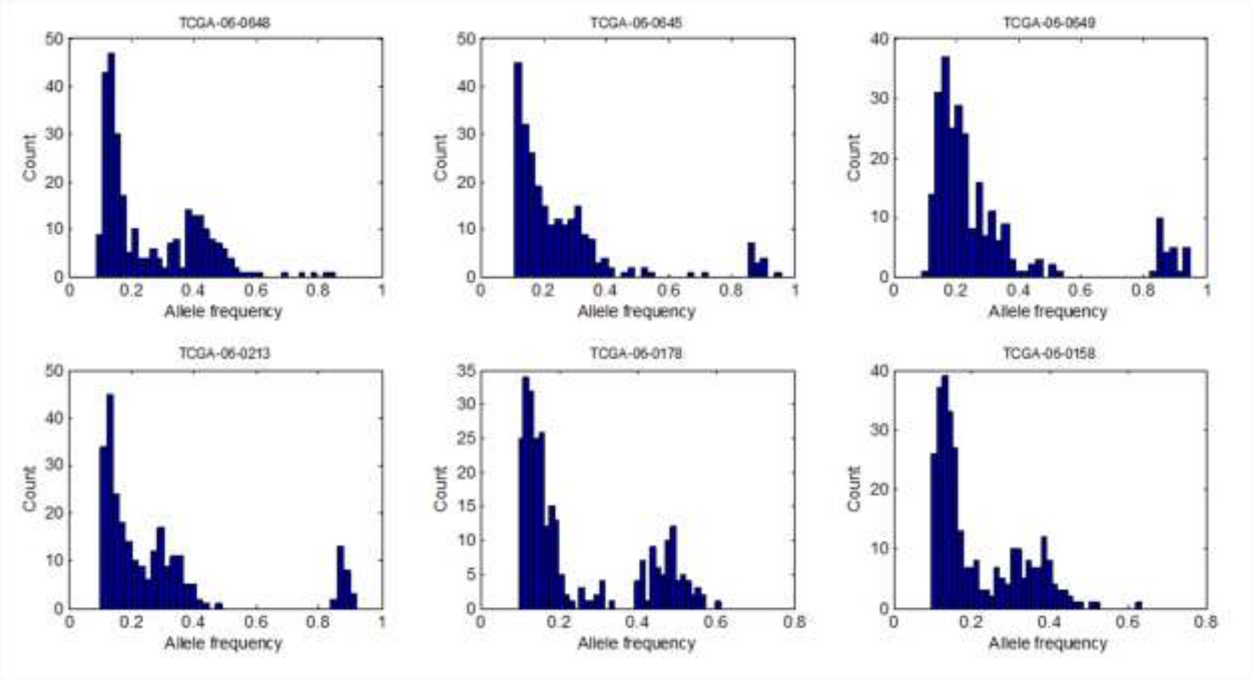

Supplementary Figure S11: Allele frequency (AF) distribution of mutations detected in six GBM exomes from TCGA. A perfectly pure tumor sample, with no normal cell contamination and one homogeneous tumor cell population is expected to have a few homozygous mutations, visible as a small peak at AF=1.0 and several heterozygous mutations visible as a large peak around AF=0.5. However since tumor-samples are rarely pure, both peaks are typically shifted to the left, on average around 0.7 and 0.35. The leftmost peak, before AF=0.2, indicate the presence of subclonal mutations or mutations within amplified regions of the genome.
